# Climate, geography and socioeconomic vulnerability and emergency care accessibility in Nepal, 2022: a high-resolution geospatial analysis of inequalities

**DOI:** 10.1136/bmjgh-2025-021150

**Published:** 2026-04-30

**Authors:** Aldina Mesic, Andrea Davis, Carlos Ochoa, Shyam Sundar Budhathoki, Raslina Shrestha, Barclay T Stewart, Christopher Millett, Thomas Hone

**Affiliations:** 1 Department of Primary Care and Public Health, School of Public Health, Imperial College London, London, UK; 2 School of Environment, Society, and Sustainability, University of Utah, Salt Lake City, Utah, USA; 3 Institute of Global Health, University of Geneva, Geneva, Switzerland; 4 Department of Burns, Plastic, and Reconstructive Surgery, Kirtipur Hospital, Kathmandu, Nepal; 5 Department of Surgery, Mayo Clinic Hospital, Rochester, Minnesota, USA; 6 Department of Surgery, University of Washington, Seattle, Washington, USA; 7 Global Injury Control Section, Harborview Injury Prevention & Research Center, Seattle, Washington, USA; 8 NOVA National School of Public Health, Public Health Research Centre, Comprehensive Health Research Center, NOVA University Lisbon, Lisbon, Portugal

**Keywords:** Injury, Nepal

## Abstract

**Background:**

Strengthening emergency care systems could reduce death and disability in low- and middle-income countries (LMICs), yet many struggle to provide timely, high-quality care. LMICs also face growing risks from climate-related shocks and mass casualty events. This study identifies unmet emergency care needs in Nepal—one of the world’s most climate-vulnerable countries—using high-resolution geospatial data to estimate socioeconomic and climate-related inequalities.

**Methods:**

We conducted a cross-sectional geospatial analysis using data from the 2022 Nepal Demographic and Health Survey (DHS), the 2021 DHS Service Provision Assessment and publicly available climate vulnerability data. Government hospitals were classified into three emergency care levels using World Health Organzation criteria: A (basic 24-hour services), B (A plus resuscitative capabilities) and C (B plus ≥50 beds and a surgeon). Household location and injury data were obtained from the DHS. Accessibility was defined as the proportion of the population within 1-hour and 2-hour travel times, estimated using AccessMod 5.8, incorporating road networks, rivers, land cover and elevation.

**Findings:**

Most public hospitals (77.7%) met Level A criteria but only 49.3% met Level B and 10.8% met Level C. Nationally, 78.7% of households had 1-hour access to Level A care, 71.8% to Level B and 44.6% to Level C. Adjusted logistic regression showed rural, poorer and climate-vulnerable households had significantly lower 1-hour access compared with urban households, rural households had lower odds of access: Level A OR: 0.33 (95%CI 0.30 to 0.37), Level B OR: 0.33 (95%CI 0.30 to 0.36), Level C OR: 0.56 (95%CI 0.51 to 0.61). Households in high climate vulnerability areas had reduced access across all levels.

**Conclusions:**

Substantial gaps and inequities in timely access to high-quality emergency care exist in Nepal. Rural, poorer and climate-vulnerable populations experience markedly lower access. Targeted, decentralised strengthening of emergency care capacity is essential both in Nepal and in other LMICs facing similar constraints.

**WHAT IS ALREADY KNOWN ON THIS TOPIC**

- Emergency care systems in many low- and middle-income countries (LMICs) are under-resourced, with facilities often lacking essential services and equipment. Prior geospatial studies in LMICs have shown significant inequalities in travel times to emergency care but most rely on facility type as a proxy for service capacity. Nationally representative analyses that integrate household-level data, facility readiness and climate vulnerability are rare, limiting our understanding of how intersecting risks affect access.

**WHAT THIS STUDY ADDS**

- This is the first nationally representative study in Nepal to combine household injury data, detailed facility-level emergency care capacity and climate vulnerability metrics. It reveals poor levels of emergency care access, particularly for rural, poorer and climate-vulnerable populations.

**HOW THIS STUDY MIGHT AFFECT RESEARCH, PRACTICE OR POLICY**

- There is an urgent need for targeted emergency care investments to address geographic and climate-related disparities. The methodology offers a replicable framework for other LMICs to assess and improve emergency care accessibility using existing national and geospatial datasets.

## Introduction

 Emergency care is vital for managing a wide range of time-sensitive conditions, including obstetric complications, acute paediatric infections, exacerbations of non-communicable diseases and injuries, where delays worsen the prognosis and reduce the effectiveness of care.^1 2^ Globally, nearly 90% of deaths and 84% of disability-adjusted life years are due to emergency conditions, with low-income countries and the African and Southeast Asia regions facing particularly high burdens.^3^ Further, climate change is expected to increase preventable injuries in the future,^4^ as the world experiences more frequent and intense disasters, challenging health systems that are already struggling to meet current emergency condition demands.

The World Health Organization (WHO) highlights the importance of integrated emergency care systems for reducing mortality and disability through essential functions such as pre-hospital care, transport and facility-based services (eg, early operative and critical care).^1 5^ Despite longstanding global commitments to strengthen care across components of the system,^6 7^ health systems in many low- and middle-income countries (LMICs) struggle to deliver accessible and timely emergency care for acute illnesses and injuries, leading to avoidable disabilities and deaths.^1 5 8^ These gaps are exacerbated by fragmented systems, workforce shortages, limited critical resources and environmental and seasonal barriers.^9 10^ Strengthening emergency care systems is a critical component of universal health coverage (UHC) and is estimated to reduce disability by 36% and mortality by 45% in LMICs.^11^

Identifying areas with the greatest demand for emergency services is essential for effective resource allocation and aligns with the World Health Assembly (WHA) Resolution 60.22 call to ‘comprehensively assess the emergency care context and identify unmet needs’.^6 7^ Despite this call, geographical analyses of emergency care access in LMICs are limited. Hospital-based capacity studies are increasing,^12^,^15^ including in Nepal,^16^ but only two account for both facility locations and the populations they cover.^8 17^ One sub-Saharan Africa study uses advanced geospatial methods but makes assumptions about which facilities should be able to provide emergency care (eg, all government hospitals) without accurately measuring capacity.^18^ This is a notable limitation, as even if populations are near facilities, this does not guarantee access, as people frequently encounter non-existent or ineffective emergency care at hospitals.^19^

Nepal is a unique case for evaluating emergency care access as the country faces substantial health system challenges, a high injury burden and vulnerability to climate disasters. Injuries are a leading cause of morbidity and mortality in Nepal,^20^ with falls, road traffic injuries and burns comprising the largest proportions of injuries.^21 22^ In 2014, 13.1% of the population reported injuries in the past year,^21^ and Nepal’s road traffic fatality rate is 28.2 per 100,000 people, one of the highest in the world.^23^ Nepal also has one of the world’s highest burn injury rates, driven by energy poverty, unsafe cooking practices and weak building enforcement. Additionally, Nepal is one of the most climate-vulnerable and disaster-prone countries in the world, with floods, landslides, earthquakes and wildfires posing the greatest threats.^24^ Such events are expected to increase in frequency and intensity due to climate change,^25 26^ necessitating the strengthening of emergency care for anticipated injuries and mass casualty events.

Nepal, like other LMICs, is focused on strengthening the emergency care system.^16^ In recent years, Nepal has invested in a national ambulance service and national trauma and burn centres and has participated in the WHO Global Emergency and Trauma Care Initiative. ^27 28^ However, there are notable challenges. Despite substantial expansion, the ambulance service still comprises only 10 ambulances nationwide, which is inadequate to meet the needs of more than 30 million people.^28^ Most emergency care is delivered by providers without formal training, as there is limited post-graduate training in emergency medicine and a shortage of specialists and positions for those specialists within government hospitals.^29^ Moreover, there is no universal emergency services number or a Good Samaritan Law to offer legal protection to bystanders and volunteers who provide care in emergencies (eg, after a road traffic collision).^27 28^ Further, Nepal’s diverse topography,^30^ dispersed population—with nearly 78% of all residents living in rural areas^31^—and persistent barriers to healthcare access^32^ compound the difficulty of delivering timely emergency care.

Uniquely among LMICs, Nepal has recent, nationally representative data capturing facility-based service availability and quality (the Demographic Health Survey (DHS) Service Provision Assessment (SPA)), the DHS household survey including injury questions and spatial measures of climate change vulnerability.^33^ These datasets offer a rare opportunity to evaluate emergency care coverage comprehensively and overcome limitations of prior studies that rely on assumed facility capacity or lack integrated population and hazard data. No prior study has combined these datasets to assess emergency care coverage in Nepal. This represents a critical gap, and addressing it could both strengthen Nepal's emergency care system and inform approaches relevant to other LMICs facing similar challenges.

Using a previously described facility-classification approach,^8^ we triangulate these data to generate the first comprehensive estimate of emergency care coverage and identify where unmet needs are greatest. Similar Geographic Information Systems (GIS) approaches (ie, cost distance analysis using AccessMod^34^) have been applied for specific care components (eg, surgery^10 35^) and conditions (eg, viral haemorrhagic fevers^36^ and snakebite^37^), but not for emergency care in a LMIC. This approach moves beyond assumptions of facility capacity, enabling the quantification of geographic, socioeconomic and climate-related inequalities. The aim of this study is to generate the first comprehensive estimate of emergency care coverage in Nepal and identify where unmet needs are greatest by integrating nationally representative facility, population and climate vulnerability data. This study demonstrates how routinely collected data can inform emergency care system strengthening, with lessons from Nepal relevant to other LMICs facing similarly constrained and hazard-exposed health systems.

## Methods

### Study setting

Nepal is a lower-middle-income South Asian country with a Gross Domestic Product of US$1377 per capita^38^ and nearly 30 million people. The country is divided into three ecological regions: mountains, hills and the Terai (plains) (see online supplemental materials), with most of the population residing in the Terai (50.27%), followed by the hills (43.1%) and mountains (6.73%).^30^ Nepal is highly vulnerable to natural disasters and climate change, especially due to extreme rainfall, heatwaves, flooding and landslides.^25^ Nepal is divided into seven provinces, 77 districts and 753 local units. Health services are delivered through a three-level system: local (primary care facilities including health posts, centres and community health units), provincial (district, zonal and provincial hospitals) and federal (central, specialised and teaching hospitals offering advanced tertiary care).

### Study design

We analysed nationally representative data from the Nepal DHS SPA 2021 and DHS 2022 to conduct a cross-sectional analysis on geographical accessibility (ie, travel time) to emergency care for the population and all DHS households (the unit of analysis) and those reporting injury in the past year using AccessMod 5.8.^34^

### Data sources

We provide a complete list and maps of spatial data sources in online supplemental table 1 and online supplemental figures A–J. For the household data, including for households reporting injuries in the past year, we accessed the Nepal DHS 2022.^39^ This national survey provides representative samples of urban and rural households on national and provincial levels. We obtained data on the location, area type (urban, rural), province, ecological zone and wealth index (a composite measure considering assets^40 41^) to quantify access inequalities across groups. Precise geospatial information is not provided with urban clusters displaced by up to 2 km and rural clusters up to 5 km.^42^ All other items on injury prevalence and characteristics of the injured person (age, sex, type of injury, type of disability) were obtained from the DHS Injury Module.

To quantify service availability and accessibility, we used the DHS SPA 2021,^43^ a nationally representative health facility census, which provides data on the quality of care in hospitals using five types of questionnaires: inventory, health worker interviews, consultation observations, exit interviews and newborn resuscitation simulations. In this analysis, we include only hospitals, as patients requiring emergency trauma care are more likely to seek services at hospitals with designated emergency units, intensive care units and operating theatres. The SPA covers all government hospitals, the focus of this study, as most Nepalis rely on public healthcare following the introduction of free essential care in 2008.^44^ A recent study found that 68% of the population sought care at public facilities, citing financial and physical accessibility as key factors. Our approach is consistent with prior research on public-sector emergency care accessibility.^18^

Other publicly available datasets required to calculate travel times were land cover from the European Space Agency,^45^ the Digital Elevation Model from the Shuttle Topography Mission,^46^ the spatial population from Meta’s High-Resolution Density Maps from the Humanitarian Data Exchange,^47^ the road network (primary, secondary, tertiary and minor roads, bridges)^48^ and rivers from OpenStreetMap.^49^ Each of these had a resolution of 30 m per pixel at the equator. To assess local climate change vulnerability, we accessed a publicly available spatial dataset of Nepal,^33^ which categorises each kilometre into a very low, low, moderate, high or very high vulnerability to climate change using a combination of 13 different biophysical and socioeconomic variables (eg, precipitation, temperature trend, irrigated land, wealth). Estimates of travel speeds and scenarios were adapted from Ochoa *et al* for four-wheelers, two-wheelers (eg, motorcycles) and three-wheelers (eg, tempos),^37^ and are included in online supplemental materials.

### Data preparation and statistical analyses

To classify emergency care capacity, we used a previously established emergency capacity classification approach^8^ adapted from the WHO’s Integrated Management for Emergency and Essential Surgical Care Toolkit, presented in online supplemental table 2. Additionally, the country-specific questions and minor adjustments to the criteria are included in online supplemental materials. We calculated descriptive statistics for hospital-level variables of province, level of hospital and area type. We present the density of facilities per population in each province.

For the household-level data, we calculated counts and proportions for categorical descriptive variables (area type, province, ecological zone, wealth index). For households which reported an injury in the past year, we calculated counts and proportions for the injured individuals across all available variables in the Injury Module including sex, age category, type of incident (either road traffic collision-related or other), type of vehicle (if road traffic collision-related), complication (ie, any disability, severe injury, death), type of injury and type of disability. For the injury and disability types, we used the country-specific responses in the DHS and did not re-code responses. We defined a severe disability as any of the following conditions: paralysis, brain damage, disfigurement, loss of limb, loss of limb function, loss of eyesight or loss of hearing. Households were mapped in ArcGIS Pro using the provided coordinates. The precise location was then overlaid with the climate change vulnerability dataset to categorise each household’s climate vulnerability category.

To quantify high-resolution service accessibility for all DHS households, we linked the datasets through several data processing steps. For all spatial datasets (online supplemental materials)*,* we changed the geographic coordinate system to a local projection for Nepal (WGS 84/UTM zone 45 N), aligned rasters to match the Digital Elevation Model, and built a merged land cover dataset accounting for land cover categories (water, trees, flooded vegetation, crops, built area, bare ground, snow/ice, rangeland), the road network and physical barriers (ie, rivers and water bodies). We developed a merged land cover which prioritised land cover types in the friction surface in the following order: primary roads, secondary roads, tertiary roads, minor roads, minor pedestrian roads, bridges, rivers and land cover (eg, trees, crops). Travel scenarios were developed based on Ochoa *et al*’s recent analysis of snakebite risk and accessibility to facilities treating snakebite syndromes in the Terai region.^37^ We used average speed for each scenario (ie, vehicle type, seasons), which are included in online supplemental materials*.*

We linked facility-level and household-level data with the merged land cover dataset to conduct an accessibility analysis using AccessMod 5.8.^34^ The analysis computed travel time surface^34^ for all DHS households and those reporting injuries to the nearest Level A, B and C hospitals,^8^ based on travel speed estimates (*see* online supplemental table 4).^37^ Ideally, emergency care would be provided within the first hour after injury (termed ‘the golden hour’),^50^ but many other prior studies^18 51^ suggest a 2-hour threshold as a more feasible benchmark for LMICs. Additional details are provided in the online supplemental materials*.*

Next, we calculate the proportion of DHS households covered within 1-hour and 2-hour travel time towards hospitals with emergency care at each respective level. We performed logistic regression analyses using 1-hour and 2-hour access indicators across all emergency care levels, with residence in urban or rural areas, household injury status, wealth groups and climate change vulnerability category as predictors. We present both regression coefficients with standard errors and odds ratios (ORs) with 95% CIs.

## Results

### Household sample characteristics

Online supplemental figure K presents the topographic profile, ecological zones and provinces of Nepal, the spatial distribution of all households sampled in the DHS (n=13908), and those that reported at least one injury in their household in the prior year (n=1426). There were no notable spatial trends or clustering in households reporting injuries compared with all sampled households. Table 1 presents characteristics for all sampled households and households reporting injury. The hills ecological zone had the highest number of households (n=6737, 48.4%) while most households fell into the lower wealth quintiles (Quintile 1 (poorest): 30.2% and Quintile 2 (poorer): 20.5%). About 19.4% (n=2658) were in the high or very high climate vulnerable areas. Households reporting injury (1426 of 13,908 households) had comparable demographic and socioeconomic profiles to all DHS households.

**Table 1:** Sample characteristics for households and households reporting injury in the past year

Variable	All DHS households† n=13 908		DHS households reporting injury† n=1426	
	n	%	n	%
Province				
Koshi	1771	12.7	228	16.0
Madhesh	2013	14.5	204	14.3
Bagmati	2171	15.6	197	13.8
Gandaki	2069	14.9	234	16.4
Lumbini	1814	13.0	138	9.7
Karnali	2286	16.4	235	16.5
Sudurpashchim	1784	12.8	190	13.3
Ecological zone				
Mountains	1276	9.2	135	9.5
Hills	6737	48.4	665	46.7
Terai	5895	42.3	626	43.9
Area type				
Urban	7264	52.2	750	52.6
Rural	6644	47.8	676	47.4
Wealth index†				
Quintile 1 (poorest)	4208	30.2	421	29.5
Quintile 2 (poorer)	2858	20.5	285	20.0
Quintile 3 (middle)	2641	19.0	300	21.0
Quintile 4 (richer)	2375	17.1	235	16.5
Quintile 5 (richest)	1826	13.1	185	13.0
Climate change vulnerability†‡				
High or very high	2658	19.4	307	21.5
Moderate	6293	45.2	633	44.4
Low or very low	4762	34.2	462	32.4
Missing	149	1.1	24	1.6

* Data from Nepal DHS 2022.

† A composite measure of a household’s cumulative living standard, which is calculated by considering assets, house building materials, and types of water/sanitation.

‡ Data from Mainali and Pricope 2017.

DHS, Demographic and Health Survey.

### Injured individuals’ sample characteristics

Online supplemental table 4 presents the characteristics of individuals who experienced a fatal or non-fatal injury. Of 1342 injured people, most were males (n=819; 61.0%), 45–59 year that is s of age (n=272; 20.3%), living in urban areas (n=710; 52.9%) and had injuries not related to road traffic collisions (n=778; 59.7%). Among those who reported a road traffic injury (n=525), motorcycles were the most common vehicle. Among those who were injured, 51.8% reported a disability, 14.3% reported a severe disability and 1.8% reported a fatality due to the injury. The most common types of injuries were cuts/bites/open wounds (n=590, 44.9%), followed by broken bones (n=389, 29.5%), whereas the most common types of disabilities were chronic pain (n=467, 50.3%), the loss of limb function (n=89, 9.6%) or multiple disabilities (n=100, 10.8%).

### Hospital sample characteristics

Table 2 presents all government hospitals and their emergency care classification. There were 148 total public hospitals, with nearly all in urban areas (n=135, 91.2%). In terms of emergency care capacity, most (n=115; 77.7%) hospitals had Level A emergency care and only half met Level B criteria (n=73; n=49.3%). Nearly all hospitals had intravenous fluids and local anaesthesia, but fewer had supplemental oxygen (n=114; 77.0%) and basic surgical instruments (n=109; 73.6%) available. Few met the criteria to be Level C hospitals (n=16; 10.8%) given a lack of sufficient numbers of overnight beds and surgeons on staff.

**Table 2:** Characteristics and emergency care classification for government hospitals

Variable	N	%	Hospitals per 100 000*
All hospitals	148	100	0.5
Province			
Koshi	24	16.2	0.5
Madhesh	13	8.8	0.3
Bagmati	37	25.0	0.6
Gandaki	22	14.9	1.0
Lumbini	22	14.9	0.4
Karnali	15	10.1	1.1
Sudurpashchim	15	10.1	0.5
Level of hospital			
Federal	25	16.9	
Provincial	77	52.0	
Local	46	31.0	
Area type			
Urban	135	91.2	
Rural	13	8.8	
Level A emergency care – hospitals with 24-hour emergency services	115	77.7	
Overnight beds	138	93.2	
At least two qualified providers†	137	92.6	
Availability of water and electricity	140	94.6	
Functioning telephone	125	84.5	
On-site latrines	142	95.9	
24-hour duty schedule	140	94.6	
Level B emergency care—hospitals with resuscitative capabilities	73	49.3	
Intravenous fluids	140	94.6	
Local anaesthesia	141	95.3	
Supplemental oxygen	114	77.0	
Basic surgical instruments‡	109	73.6	
Level C emergency care—‘tertiary’ hospitals§	16	10.8	
50 or more inpatient beds	35	23.6	
Surgeon on staff	40	27.0	

* The populations are as follows: Nepal: 29 159 913; Bagmati: 6 633 331; Gandaki: 2 214 094; Karnali: 1 414 913; Koshi: 5 243 137; Lumbini: 4 570 884; Madhesh: 6 050 574; Sudurpashchim: 3 032 980; Hills: 11 983 037; Mountains: 1 613 158; Terai: 15 533 097.

† Qualified providers are defined as specialist physicians, medical officers or nurses.

‡ Basic surgical equipment refers to forceps, a needle driver and sterile scissors.

§ The survey does not designate tertiary/referral hospitals, so we used the latter definition (all B criteria, 50 inpatient beds and a surgeon on staff).

DHS, Demographic and Health Survey.

### Emergency care access

**Figure 1:**
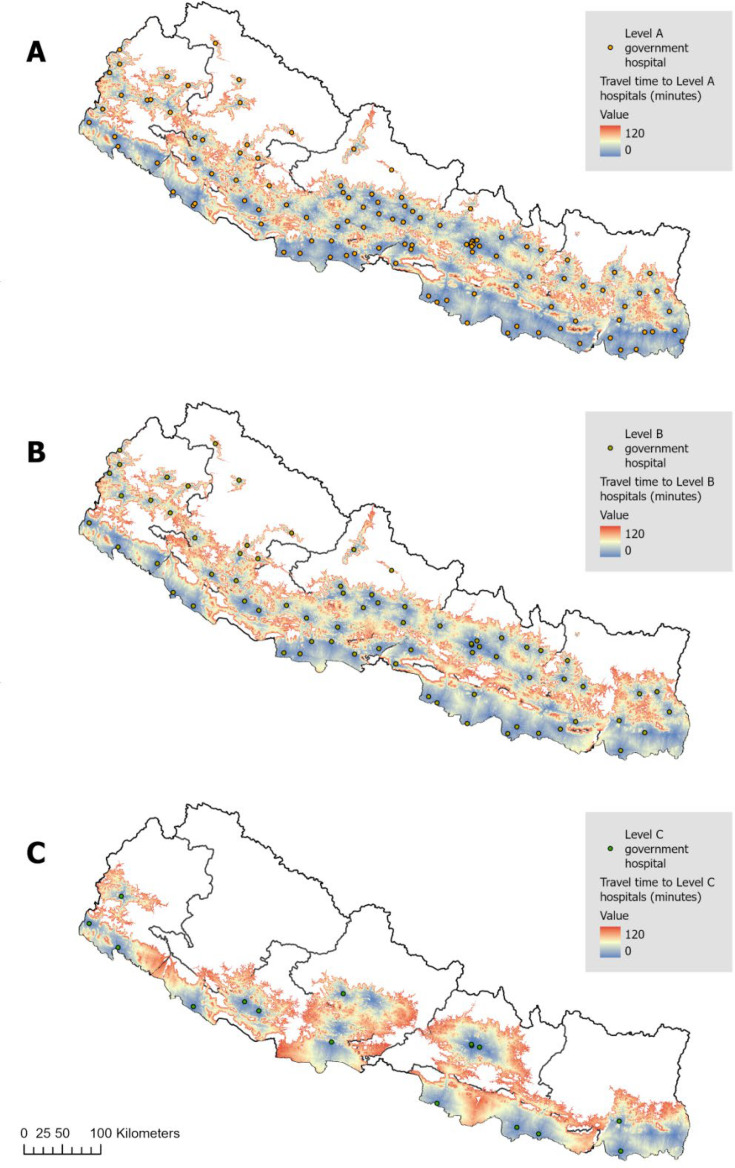
Maps of the travel time to Level A, B and C government hospitals. (A) Travel time towards Level A emergency care (hospitals with 24-hour emergency services); (B) travel time towards Level B emergency care (hospitals with resuscitative capabilities); (C) travel time towards Level C emergency care (hospitals with resuscitative capabilities, ≥50 beds and a surgeon).

Figure 1 presents travel time to access facilities meeting the three levels of emergency care capacity with a four-wheel vehicle during the dry season. As demonstrated in the figure, there are spatial patterns in access, particularly for Level C hospitals. We provide summary statistics for the DHS households and households reporting injury that have access to Level A, B and C hospitals in under 1 hour in table 3*,* whereas the 2-hour results are provided in the online supplemental materials*.* Nationally, 78.7% of DHS households have 1-hour access to Level A care, 71.8% to Level B and 44.6% to Level C. There are notable provincial differences, with Karnali consistently demonstrating the poorest access across levels (47.8% to Level A, 34.8% to Level B, and 3.3% to Level C) compared with other provinces. Households in the hills’ region had the highest access to Level B (n=3526, 84.4%) and Level C (n=2477, 59.3%), while households in the mountains’ region had the highest access to Level A (89.4% compared with 77% for hills and 72.7% for Terai). For Level C care, the Terai region has the lowest proportion of people living within 1 hour at 34.5%. Rural households are disadvantaged across all levels. For example, only 31.2% of rural households were within 1 hour of Level C, compared with 56.8% of urban households. Observationally, these estimates and patterns align across households reporting an injury at national, provincial and urban/rural levels.

**Table 3:** High-resolution physical access to government hospitals in under 1 hour

	Level A				Level B				Level C			
	All DHS households n=13 908		DHS households reporting injury n=1426		All DHS households n=13 908		DHS households reporting injury n=1426		All DHS households n=13 908		DHS households reporting injury n=1426	
	n	%	n	%	n	%	n	%	n	%	n	%
National	10 950	78.7	1120	78.5	9986	71.8	1011	70.9	6201	44.6	625	43.8
Province												
Koshi	1671	77.0	155	78.7	1468	67.6	134	68.0	1175	54.1	116	58.9
Madhesh	2069	100.0	234	100.0	2039	98.6	227	97.0	1392	67.3	158	67.5
Bagmati	1843	80.6	190	80.9	1662	72.7	173	73.6	1085	47.5	106	45.1
Gandaki	1560	86.0	120	87.0	1324	73.0	101	73.2	783	43.2	58	42.0
Lumbini	1750	86.9	171	83.8	1667	82.8	160	78.4	1024	50.9	97	47.5
Karnali	847	47.8	108	47.4	616	34.8	74	32.5	59	3.3	8	3.5
Sudurpashchim	1210	67.8	142	74.7	1210	67.8	142	74.7	683	38.3	82	43.2
Ecological zone												
Terai	5367	72.7	541	71.2	4817	65.3	477	62.8	2549	34.5	245	32.2
Hills	3737	77.0	410	90.1	3526	84.4	386	84.8	2477	59.3	264	58.0
Mountains	1671	89.4	155	78.7	1468	67.6	134	68.0	1175	54.1	116	58.9
Type of area												
Urban	6601	90.9	676	90.1	6277	86.4	631	84.1	4128	56.8	399	53.2
Rural	4349	65.5	444	65.7	3709	55.8	380	56.2	2073	31.2	226	33.4
Wealth index*												
Q1(Poorest)	2025	48.1	186	44.2	1599	38.0	141	33.5	588	14.0	56	13.3
Q2 (Poorer)	2337	81.8	241	84.6	2052	71.8	208	73.0	1151	40.3	114	42.3
Q3 (Middle)	2467	93.4	280	93.3	2305	87.3	262	87.3	1516	57.4	173	57.7
Q4 (Richer)	2319	97.6	232	98.7	2232	94.0	219	93.2	1552	65.3	152	64.7
Q5 (Richest)	1802	98.7	181	97.8	1798	98.5	181	97.8	1394	76.3	130	70.3
Climate change vulnerability†												
High or very high	1349	49.9	154	50.2	1141	42.2	124	40.4	414	15.3	52	16.9
Moderate	4897	85.2	494	78.0	4344	69.0	435	68.7	2234	35.5	218	34.4
Low or very low	4555	95.7	448	97.0	4352	91.4	428	92.6	3434	72.1	336	72.7

* A composite measure of a household’s cumulative living standard, which is calculated by considering assets, house building materials, and types of water/sanitation.

† Data from Mainali and Pricope 2017.

DHS, Demographic and Health Survey.

Climate vulnerability and wealth quintiles follow similar patterns, with the poorer and more vulnerable populations demonstrating considerably poorer access. Compared with the highest wealth quintile group, the lowest shows substantially lower 1-hour access at all levels: 51% lower for Level A (48.1% vs 98.7%), 61% lower for Level B (38.0% vs 98.5%) and 82% lower for Level C (14.0% vs 76.3%). Households in the highest category of climate change vulnerability had the poorest access to the highest level of care. For Level C hospitals, only 15.3% of households in the high or very high climate change vulnerability areas had 1-hour access, compared with 35.5% in the moderate areas and 72.1% in the low or very low areas.

**Figure 2:**
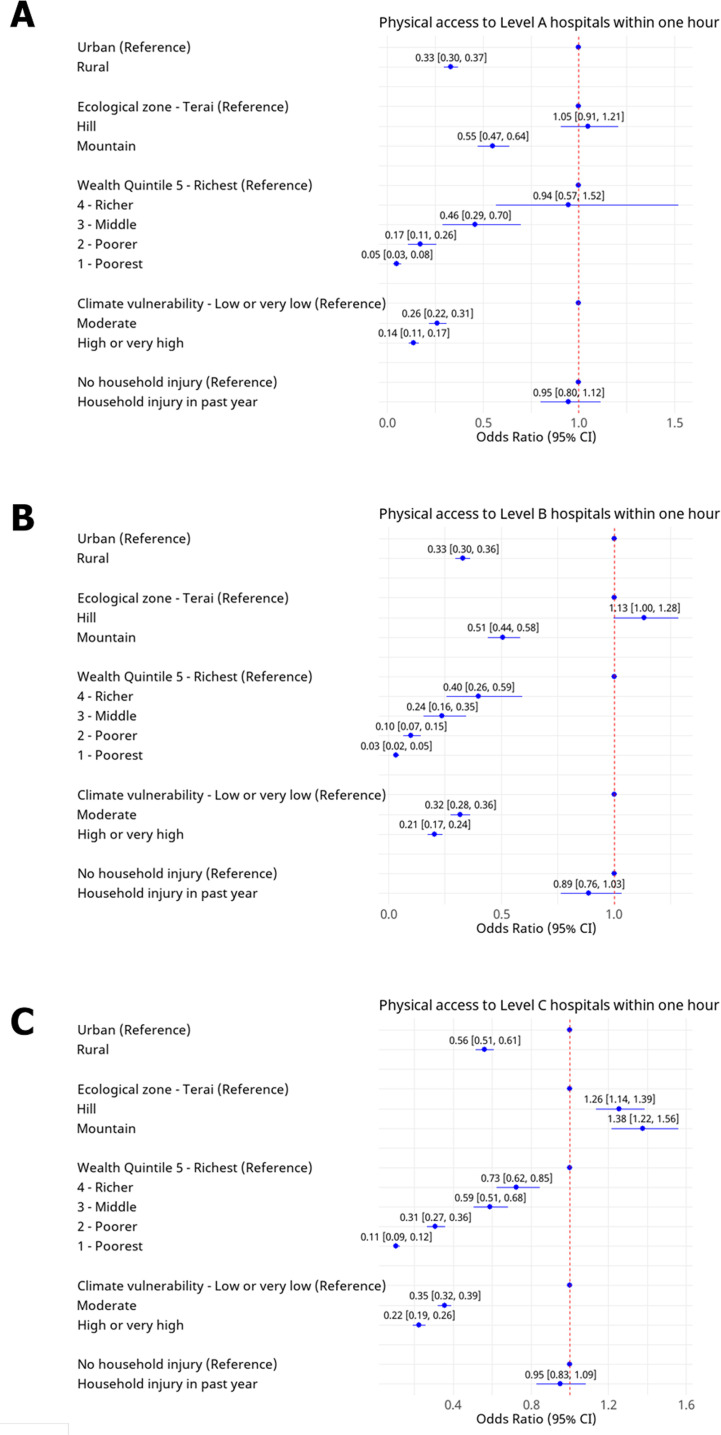
Logistic regression results for 1-hour access to government hospitals (A) 1-hour access to Level A emergency care (hospitals with 24-hour emergency services); (B) 1-hour access to Level B emergency care (hospitals with resuscitative capabilities); (C) 1-hour access to Level C emergency care (hospitals with resuscitative capabilities, ≥50 beds and a surgeon).

In adjusted logistic regression models assessing access within 1 hour to emergency care, households that were rural, poorer and in more climate vulnerable areas had consistently lower levels of access (figure 2; online supplemental figure M presents 2-hour access results). Households in rural areas are significantly less likely to have access to emergency care at all levels compared with those in urban areas (Level A OR: 0.33 (95% CI 0.30 to 0.37); Level B OR: 0.33 (95% CI 0.30 to 0.36); and Level C OR: 0.56 (95% CI 0.51 to 0.61). Poorer households demonstrate consistently lower 1-hour access compared with wealthier groups. For example, 1-hour access to Level C hospitals was significantly lower for the lowest wealth quintile group compared with the highest, with an OR of 0.11 (95% CI 0.09 to 0.12). Similarly, across climate change vulnerability categories, households in the very high or high climate change vulnerability demonstrate lower 1-hour access across all levels of care (Level A OR: 0.14 (95%CI 0.11 to 0.17), Level B 0.21 (0.17 to 0.24), Level C 0.22 (0.19 to 0.26)) when compared with the low or very low climate group.

## Discussion

Our study employed high-resolution geospatial analysis to quantify household access to three levels of emergency care capacity at government hospitals. Nearly all public hospitals were in urban areas and more than three-quarters met the minimum requirements for basic Level A emergency, yet only half of the hospitals were Level B (resuscitation-capable) and only 10% of hospitals had the highest level of emergency care (Level C). Approximately 79% of the population live within a 1-hour drive to a Level A facility while only 45% lived within 1 hour of a Level C facility. There were strikingly large inequalities in access to emergency care, which persist after adjusting for other factors. Rural populations, poorer households and those in areas most vulnerable to climate change had the lowest timely access to emergency care. Rural households demonstrated about two-thirds lower access to Level A and Level B, and half of the access to Level C, compared with their urban counterparts. Poorest households had nearly 90% lower access to all levels of care, whereas households living in areas with high climate vulnerability were nearly five times less likely to have timely access to the highest level of emergency care, compared with those in low climate vulnerability areas. These findings highlight how the intersection of geographic, socioeconomic, and environmental vulnerabilities compound to limit access to critical emergency care services.

The major gaps in the provision of emergency care identified in this study align with previous research on unmet surgical and emergency care needs in Nepal.^16 22^ Certain emergency criteria (eg, latrines, water, electricity, intravenous fluids, local anaesthesia) were widely available in hospitals. However, elements critical for resuscitation and surgical interventions (supplemental oxygen, surgical instruments, a surgeon) were not universally present, highlighting gaps for treating time-sensitive conditions. This finding aligns with recent work from hospitals in Kathmandu demonstrating that public governmental hospitals struggle to provide essential trauma care.^16^ This is particularly troubling given these hospitals are likely to be the first point of emergency care given limited pre-hospital services.^28^ Our findings confirm that few hospitals have a surgeon on staff, which aligns with prior studies noting a lack of trained personnel^52^ and specialists.^29^

The patterns of injury identified in this study showed that older men in urban areas were most likely to be injured. The most common cause of injury was ‘non-road traffic collisions’, although the survey did not specify the other causes. Other studies show that falls are the most common mechanism of injury,^21^ followed by road traffic injuries^20^ and other causes (eg, burns and poisonings^53^).

Among those injured in road traffic collisions, powered two-wheelers were most commonly involved, aligning with WHO estimates that powered two-wheelers and three-wheelers make up 34% of road traffic fatalities in Nepal.^23^ We did not find any notable differences in access between the households that reported injuries and all households, indicating that inequalities (in terms of geography, economic status and climate vulnerability) are persistent and systemic, not only for those experiencing injury.

The finding that rural and poorer households had the poorest access to emergency care is unsurprising yet is consistent with prior studies.^54^,^56^ Geographical distance and financial barriers to effective transport are likely to explain these findings. Similar socioeconomic patterns in rurality and wealth have been documented in other LMICs, such as in Ghana, where poorer households face barriers to surgical care.^54^

This study found that Nepalese populations in the highest-risk climate change areas were nearly five times less likely to live within 1 hour of any level of emergency care than less vulnerable populations. The magnitude of these inequalities was particularly worrying given Nepal’s commitment to advancing UHC and its future as a highly climate-vulnerable country. No studies to date have robustly assessed access to emergency care by area of climate vulnerability in an LMIC. In our study, climate change vulnerability was persistently associated with poorer emergency care access, even after adjusting for ecological zones, rurality and wealth, suggesting that climate vulnerability was independently associated with poor access and highlighting the vulnerability of these households. A recent systematic review in Nepal highlighted that poor and marginalised populations are most impacted by climate change, including food insecurity, drought, and floods.^57^ Globally, climate-vulnerable populations face multiple risks due to occupational exposures (eg, working outdoors in agriculture, poor housing conditions)^4^ and are often excluded from adaptation and mitigation strategies.^57^

In line with the global commitments such as the WHA Resolution 60.22, the findings from this research underscore the need for routine monitoring of emergency and surgical care capacity. Such monitoring can identify unmet needs and strengthen capacity at first-referral hospitals, which is critical to achieving UHC.^6 7^ We advocate for a targeted and decentralised approach to investment in emergency care capacity which acknowledges the unique geographical, economic and climate-related vulnerability of populations in need. For example, Karnali province and the Terai ecological zone would benefit from investment into a larger public facility with sufficient bed capacity and trained surgical staff. Alternative strategies, such as enhanced pre-hospital systems, mobile emergency units, or trained layperson first responders^58^ could address challenges related to accessibility, such as the difficult terrain and inadequate road networks in certain areas. The intersection of climate vulnerability and poor health service access emphasises the urgency of integrating emergency preparedness into climate adaptation and disaster planning, particularly for extreme weather events such as floods, landslides, wildfires and earthquakes, as well as household and individual responses to extreme weather (eg, burns from warming and frostbite^59^).

Our limitations should be noted and include assumptions about capacity, travel speeds and accessibility. For emergency care, we relied on a classification system published in 2015,^8^ which may not fully reflect the ability of a facility or its staff to manage time-sensitive conditions in a high-quality manner. Other approaches, such as an Essential Package of Emergency Care and availability of essential functions,^1^ have been proposed, but cannot be measured using the existing SPA questionnaire, which lacks a dedicated emergency care module. Relatedly, our analysis is limited to government hospitals. Although most individuals seek care in public facilities, this approach may underestimate access to private hospitals offering comparable, or better, services. A prior study found that among the roughly 32% who first seek care in private facilities, most do so because they perceive better resources and healthcare delivery.^44^ However, it is unlikely that private hospitals would be available in rural, poorer and climate-vulnerable areas, and therefore this is unlikely to negate our findings. For travel speeds, we relied on estimates from a study in the Terai region, which may overestimate speeds in the hills and mountains, although elevation was considered in the accessibility model to address this concern.^37^ We applied both 1-hour and 2-hour accessibility to be consistent with established benchmarks^18^ while noting that shorter times may reduce adverse outcomes for those seeking care after an injury. Our maps show results from 0 to 2 hours to offer a more nuanced understanding of coverage and accessibility. Our main estimates are during the dry season, and accessibility may be lower during the wet seasons.

Some limitations stem from the DHS. First, for quantifying accessibility, we assume that the injury occurred at the place of residence, as the location was not recorded. Relatedly, some data were missing from the injury module, including the mechanism of injury for non-road-traffic injuries, and precise definitions for incident types, injuries and disabilities were not provided. Further, we note that the household data were collected during the COVID-19 pandemic, and this impacted the data collection^60^ and the observed patterns of injury (eg, increased injuries at home due to lockdowns,^61^ fewer road traffic injuries).^62^ In Nepal, a recent study confirmed that road traffic collisions, injuries and deaths decreased during the COVID-19 lockdown, but that this reduction was smaller than expected due to higher vehicle speeds due to relatively lower traffic volumes and limited enforcement.^63^

Second, households are displaced to protect the confidentiality of households in the DHS data. While this limitation is well-documented,^42^ it introduces uncertainty. Other analyses have linked households and hospitals using administrative areas, Euclidean buffers,^64 65^ road networks and kernel density estimation,^66^ but found that all result in potential misclassification error^66^ and do not account for key geographic factors influencing access (eg, natural barriers).^10^ In contrast, our analysis computes travel time surfaces by considering travel speeds, the road network, land cover and barriers, factors that are critical to understanding real-world accessibility.

Nonetheless, there are key strengths to our work. Our study demonstrates how high-quality, nationally representative spatially linked data can be used to understand inequalities in emergency care capacity and accessibility—a first for an LMIC. We employ validated methods for linkage and geospatial analysis and test the validity of our findings with robustness checks. While the future of the DHS Programme is uncertain,^67^ our analysis showcases its potential as more countries gather data on injuries and assess health service quality through the SPA to inform targeted emergency care capacity strengthening.

## Conclusion

Major gaps in physical access to high-quality, life-saving emergency healthcare exist in Nepal, reflecting the broader challenges faced by many LMIC emergency care systems. Our analysis shows that although most public hospitals meet basic emergency care requirements, only half are resuscitation-capable and just 1 in 10 provides the highest-level emergency services. We find that less than half of the population live within 1 hour of hospitals equipped to the highest standard. Proximity to facilities does not guarantee access to care, as many hospitals lack the critical resources needed for effective emergency treatment. These gaps are concerning because emergency conditions account for the vast majority of global mortality and disability, and delays in care significantly worsen outcomes.

Rural and poorer households, as well as those in climate-vulnerable areas, have substantially lower access to emergency care, highlighting persistent inequities in the health system. The intersection of geographic, socioeconomic and climate-related vulnerabilities leaves the populations at greatest risk with the least timely access to effective care. By integrating nationally representative facility, household and climate vulnerability data, this study provides the first comprehensive assessment of emergency care coverage in Nepal and identifies where unmet needs are greatest. Strengthening emergency care is essential for advancing UHC in one of the world’s most climate-vulnerable countries and offers lessons for other LMICs confronting similar constraints.

Future research should explore the quality, timeliness and availability of prehospital emergency care in Nepal and other LMICs. Incorporating data on seasonal weather patterns and climate events could improve emergency preparedness for both routine care and mass casualty incidents. Studies evaluating specific barriers, such as workforce shortages and limited bed capacity, are needed to guide capacity-building efforts. Finally, as targeted emergency care strengthening investments are made, assessing their impact on reducing inequities will be critical.

## Supplementary material

10.1136/bmjgh-2025-021150 online supplemental file 1

10.1136/bmjgh-2025-021150 online supplemental file 2

## Data Availability

Data are available in a public, open access repository.
