# Supplementary material for: Climate, geography and socioeconomic vulnerability and emergency care accessibility in Nepal, 2022: a high-resolution geospatial analysis of inequalities
Source: BMJ Glob Health. 2026 Apr 30;11(4):e021150. doi: 10.1136/bmjgh-2025-021150 (PMC13141136; doi:10.1136/bmjgh-2025-021150)
Supplement: online supplemental file 1 [file bmjgh-11-4-s001.docx]

**Supplementary Materials**

[Table 1: Spatial data sources 2](#_Toc218850360)

[Appendix 1: Data processing and statistical analyses - additional information 3](#_Toc218850361)

[Figure A: Map of government hospitals from the Nepal DHS Service Provision Assessment 2021 4](#_Toc218850362)

[Figures B & C: Map of households and households reporting injury in the past year from the Nepal DHS 2022 4](#_Toc218850363)

[Figure D: Map of land cover from the European Space Agency 5](#_Toc218850364)

[Figure E: Map of the Digital Elevation Model from the Shuttle Topography Mission 5](#_Toc218850365)

[Figure F: Map of Spatial Population from Meta’s High Resolution Density Maps 6](#_Toc218850366)

[Figure G1: Map of primary roads from Open Street Map 6](#_Toc218850367)

[Figure G2: Map of secondary roads from Open Street Map 7](#_Toc218850368)

[Figure G3: Map of tertiary roads from Open Street Map 7](#_Toc218850369)

[Figure G4: Map of minor roads from Open Street Map 8](#_Toc218850370)

[Figure H: Map of rivers from Open Street Map 8](#_Toc218850371)

[Figure I: Map of Climate Vulnerability from Mainali and Pricope (1) 9](#_Toc218850372)

[Figure J: Map of bridges from Open Street Map 9](#_Toc218850373)

[Table 2: Emergency capacity classification criteria (adapted from Tansley et al., 2015)(3) 10](#_Toc218850374)

[Table 3: Nepal SPA 2021 – Facility questionnaire (NPFC8AFLSR) mapped to emergency care criteria 11](#_Toc218850375)

[Table 4: Average speeds, all vehicle types 12](#_Toc218850376)

[Table 5: Sample characteristics for injured people 13](#_Toc218850377)

[Table 6: High-resolution physical access to government hospitals in under two hours 14](#_Toc218850378)

[Figure K: Map of topography, provinces, ecological zones, sampled households, and households reporting injury in the past year 15](#_Toc218850379)

[Figure L: Maps of the spatial distribution of Level A, B, and C government hospitals 16](#_Toc218850380)

[Figure M: Logistic regression results for two-hour access to government hospitals 17](#_Toc218850381)

[References 18](#_Toc218850382)

## Table 1: Spatial data sources

| Data | Source |
| --- | --- |
| 1. Location and emergency care capacity of government hospitals | Nepal DHS Service Provision Assessment 2021 |
| 1. Demographic data (area type, province, wealth index) and location of households | Nepal DHS 2022 |
| 1. Injury prevalence and characteristics data | Nepal DHS 2022, Injury module |
| 1. Land cover | European Space Agency |
| 1. Digital Elevation Model (DEM) | The Shuttle Topography Mission |
| 1. Total Spatial Population | Meta’s High Resolution Density Maps from the Humanitarian Data Exchange |
| 1. Road network | Open Street Map |
| 1. River network | Open Street Map |
| 1. Climate change vulnerability | Mainali and Pricope 2017 (1) |
| 1. Bridges | Open Street Map |

## Appendix 1: Data processing and statistical analyses - additional information

To quantify high-resolution service accessibility for all DHS households, we linked the datasets through several data processing steps. First, we changed the geographic coordinate system to a local projection for Nepal (WGS 84/UTM zone 45 N) in all datasets, aligned rasters to match the Digital Elevation Model, and built a merged land cover dataset accounting for land cover categories (water, trees, flooded vegetation, crops, built area, bare ground, snow/ice, rangeland), the road network, and physical barriers (i.e., rivers and water bodies). We developed a merged land cover which prioritised land cover types in the friction surface in the following order: primary roads, secondary roads, tertiary roads, minor roads, minor pedestrian roads, bridges, rivers, and land cover (e.g., trees, crops). Travel scenarios were developed based on Ochoa *et al.*’s recent analysis of snakebite risk and accessibility to facilities treating snakebite syndromes in the Terai region (2). We used average speed for each scenario (i.e., vehicle type, seasons), which are included in *Supplementary Materials.*

While the OSM road network and Ochoa *et al.* included several road classes (e.g., trunk, service), we simplified roads into four categories: 1) minor roads (road, residential, living street, service, track, unclassified, bridleway, pedestrian, path, footway, cycleway); 2) tertiary roads (tertiary, tertiary link); 3) secondary roads (secondary, secondary link); 4) primary roads (trunk, trunk link, primary).

To acknowledge the influence of elevation, we selected the anisotropic option for the accessibility analysis, which uses the Digital Elevation Model to calculate the slope of the terrain and adjust for up- and downhill walking speeds. The resulting outputs are raster files showing two-hour/120-minute travel time catchment areas used to calculate the proportion of the population with physical access. For each emergency capacity level (A, B, and C) analysis, we selected four-wheeler vehicles during the dry season with 120-minute travel time as the default scenario. In sensitivity analyses, we repeated this analysis during the wet season and for other vehicle types (e.g., motorcycles, powered three-wheelers), finding similar results due to the travel scenarios and cut-off decision. Therefore, the results are not presented.

## Figure A: Map of government hospitals from the Nepal DHS Service Provision Assessment 2021


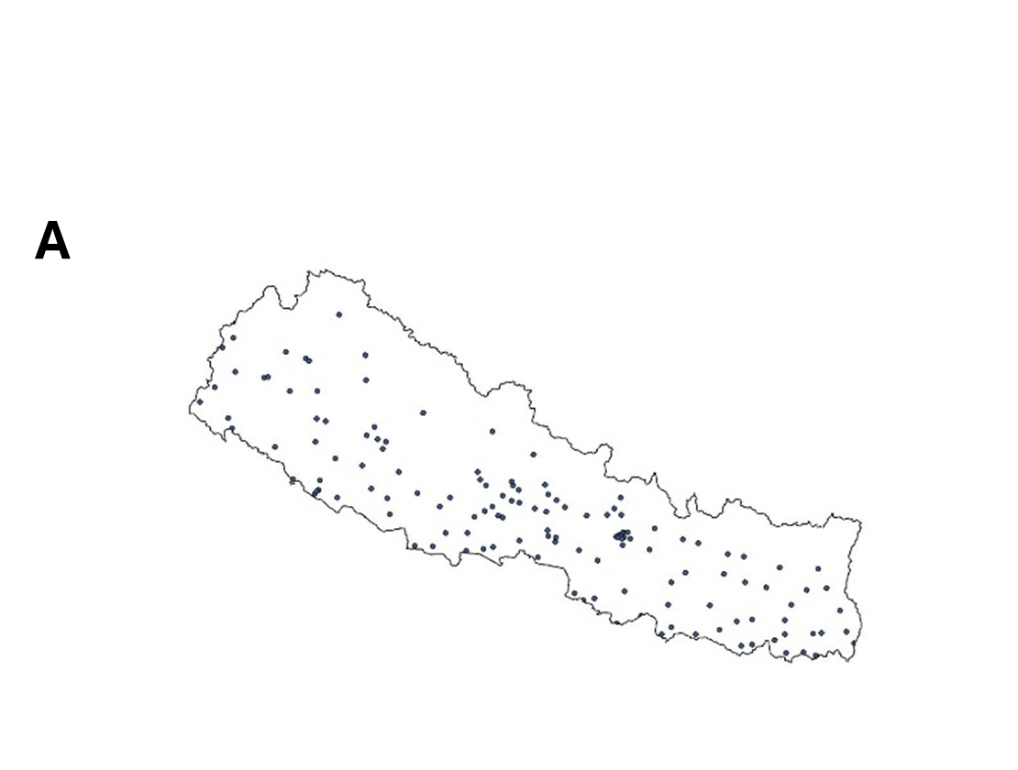


## Figures B & C: Map of households and households reporting injury in the past year from the Nepal DHS 2022


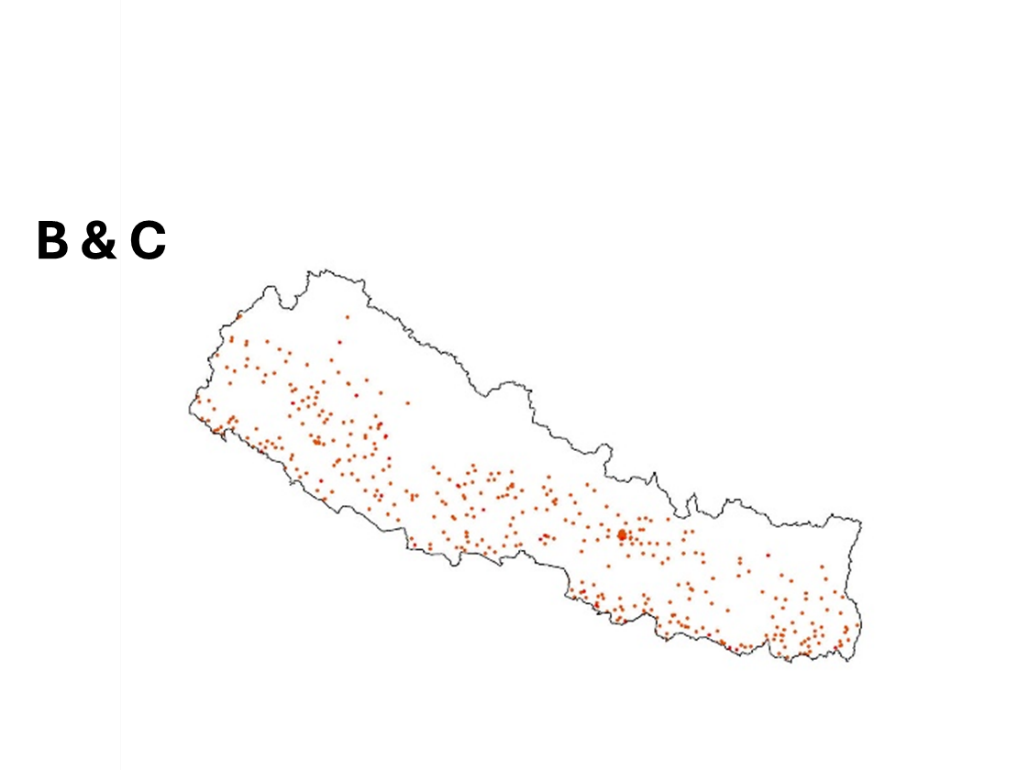


## Figure D: Map of land cover from the European Space Agency


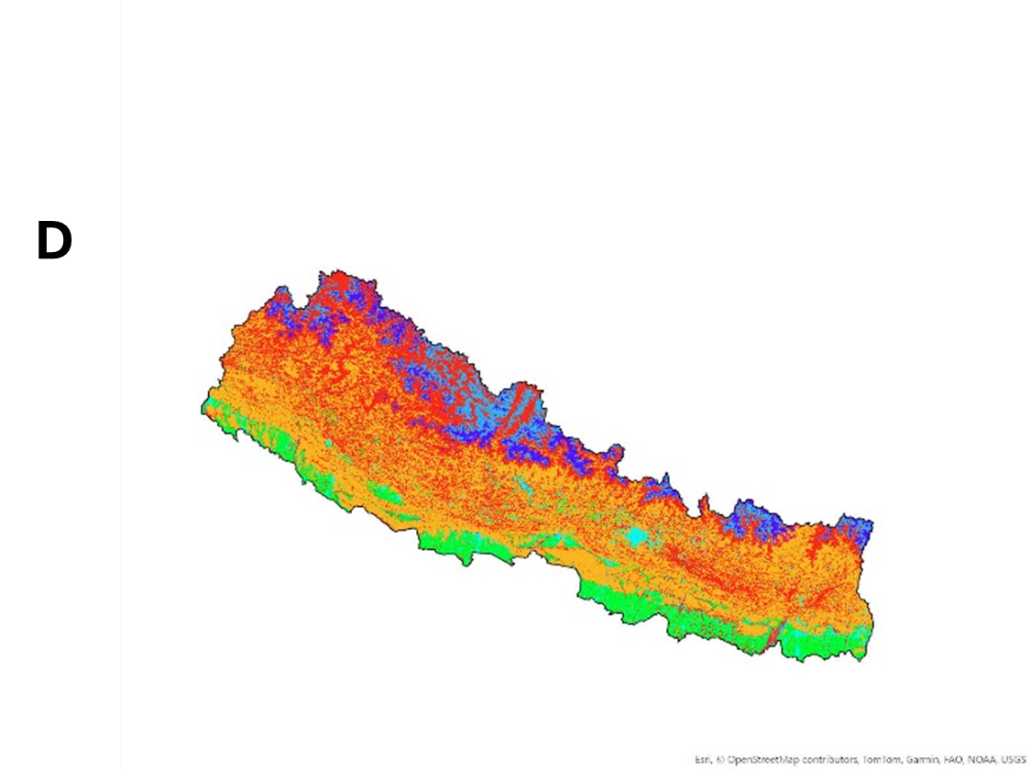


## Figure E: Map of the Digital Elevation Model from the Shuttle Topography Mission


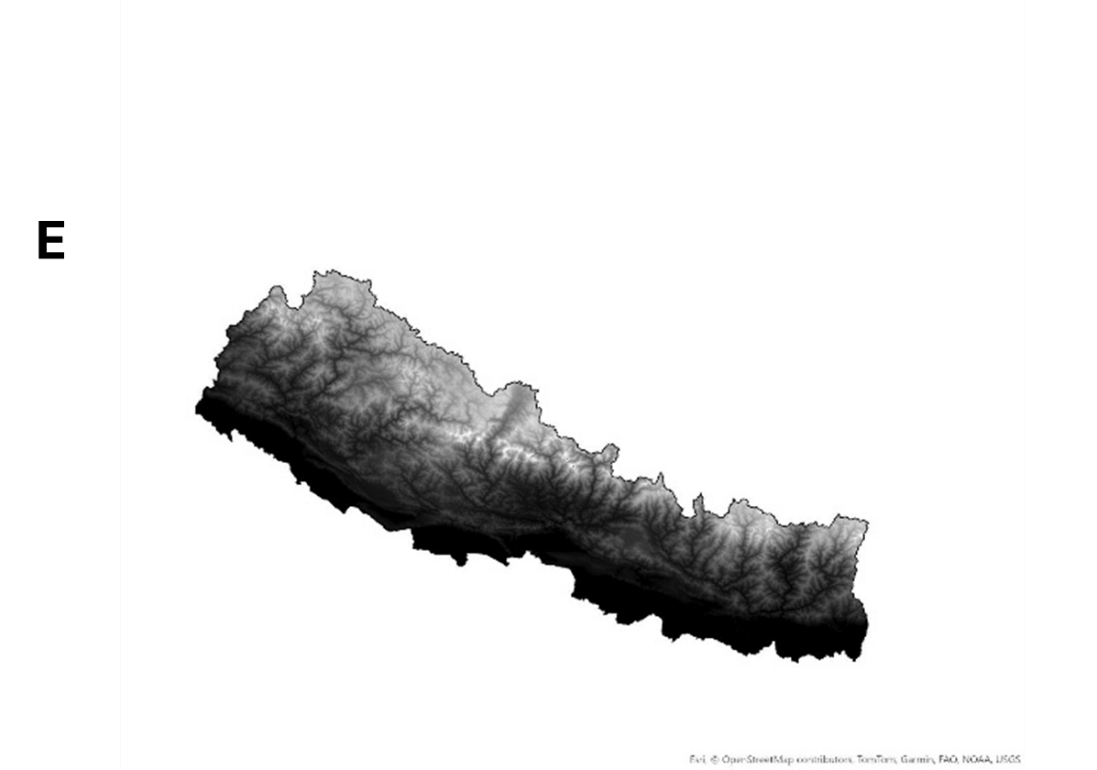


## Figure F: Map of Spatial Population from Meta’s High Resolution Density Maps


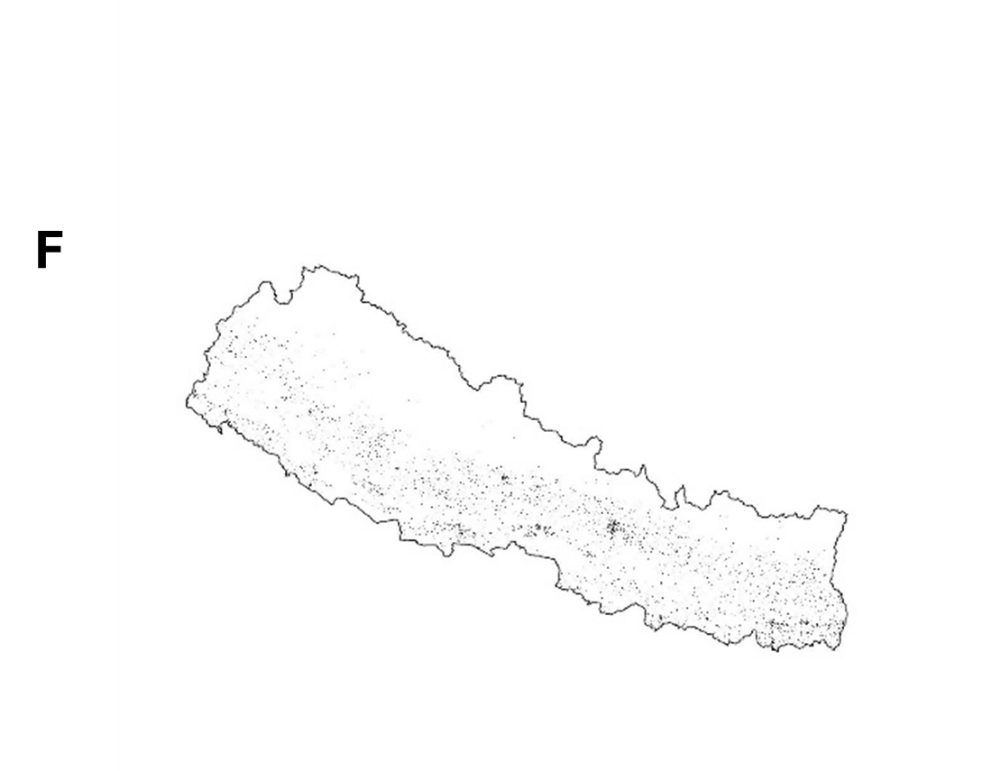


## Figure G1: Map of primary roads from Open Street Map


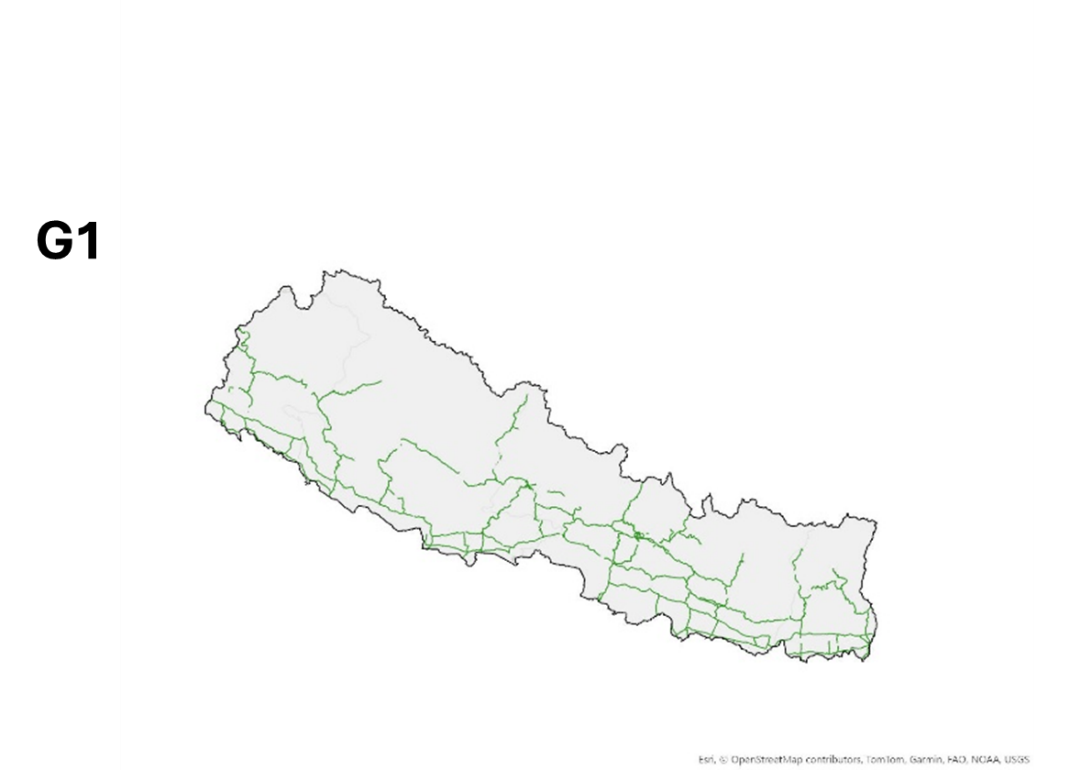


## Figure G2: Map of secondary roads from Open Street Map


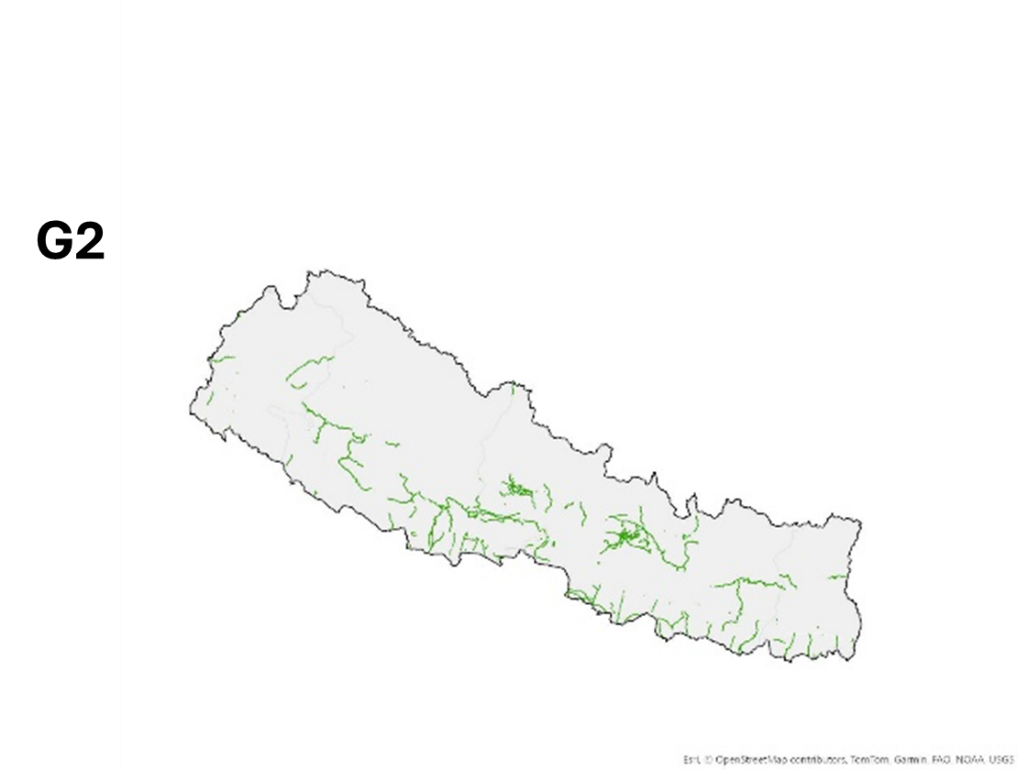


## Figure G3: Map of tertiary roads from Open Street Map


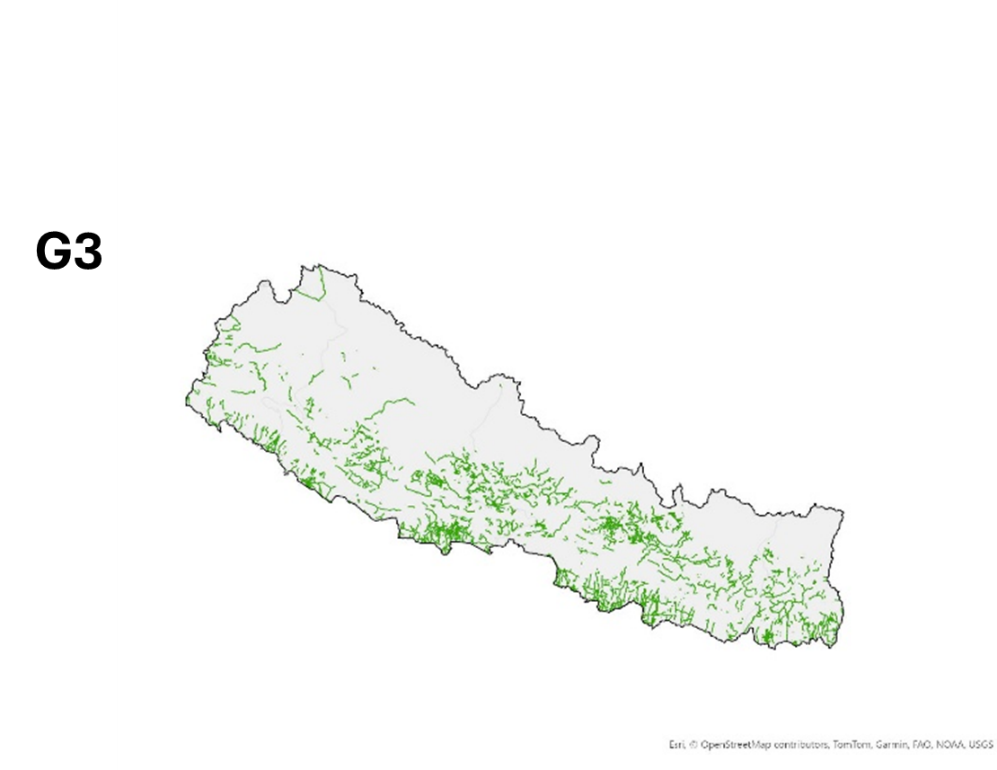


## Figure G4: Map of minor roads from Open Street Map


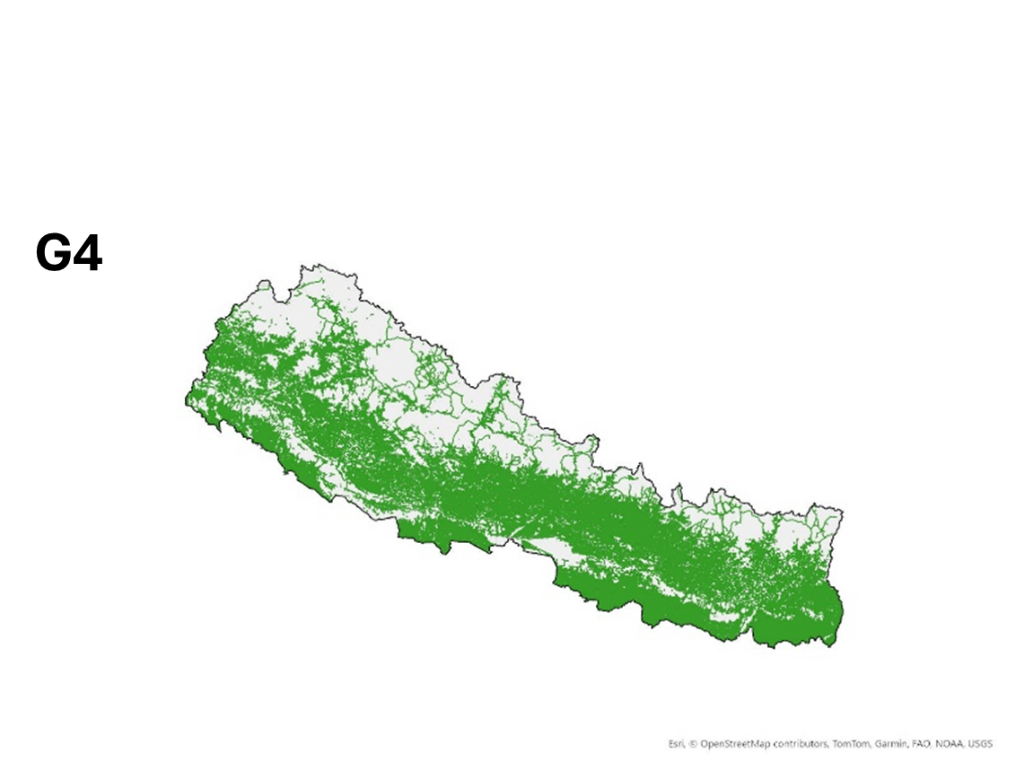


## Figure H: Map of rivers from Open Street Map


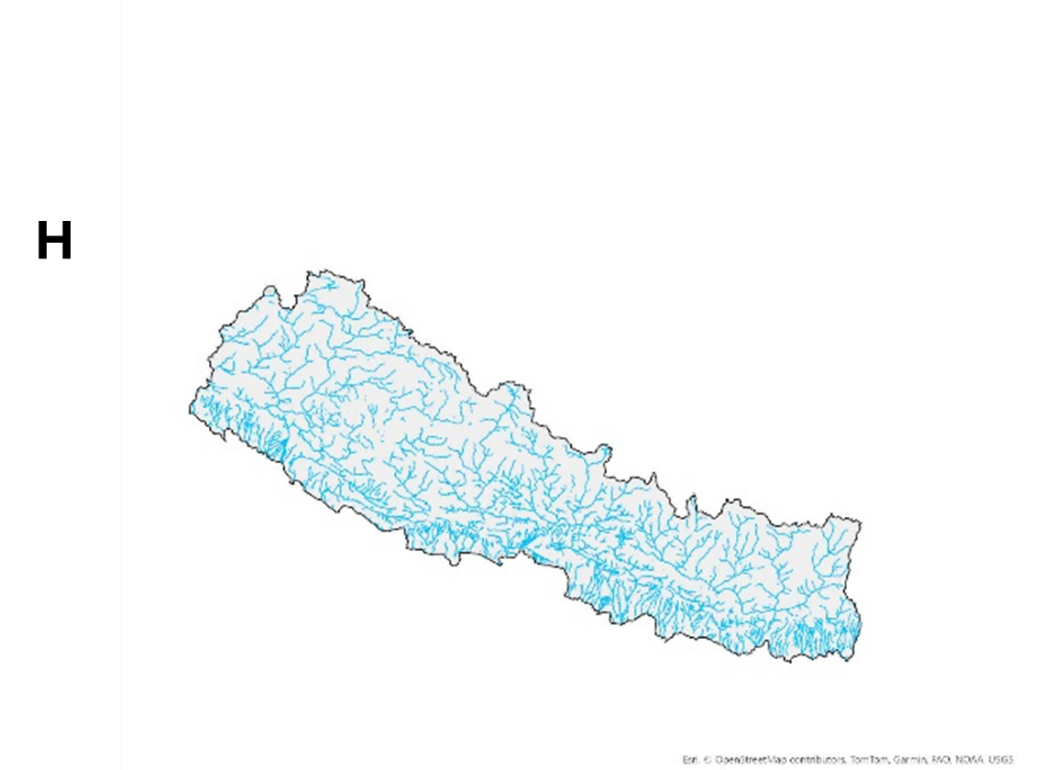


## Figure I: Map of Climate Vulnerability from Mainali and Pricope (1)


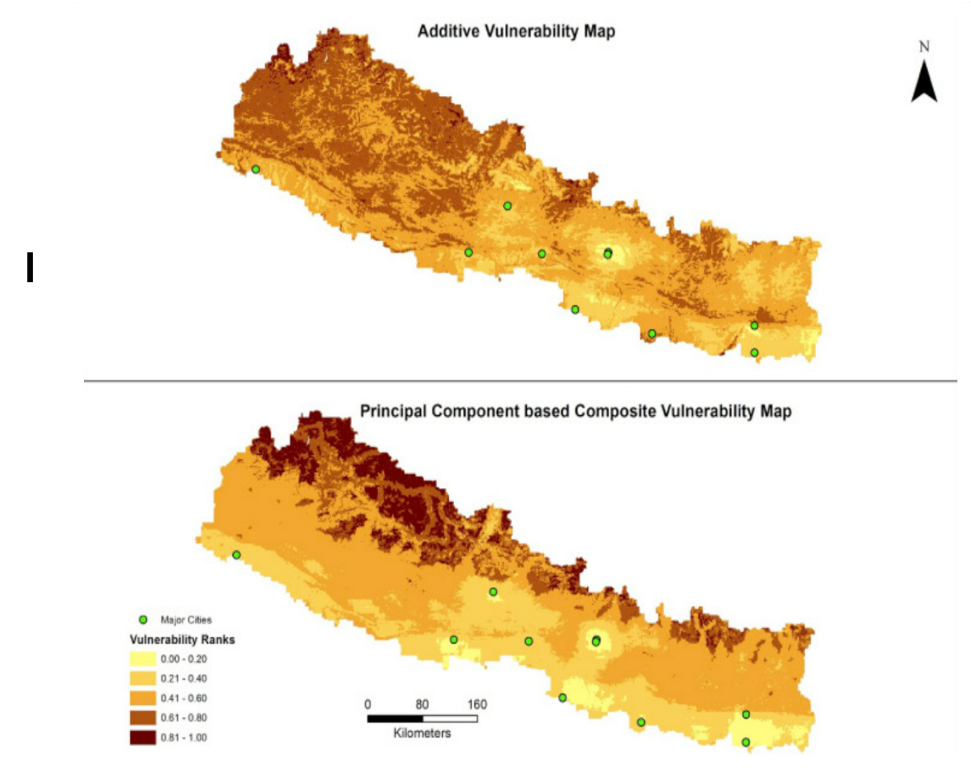


## Figure J: Map of bridges from Open Street Map


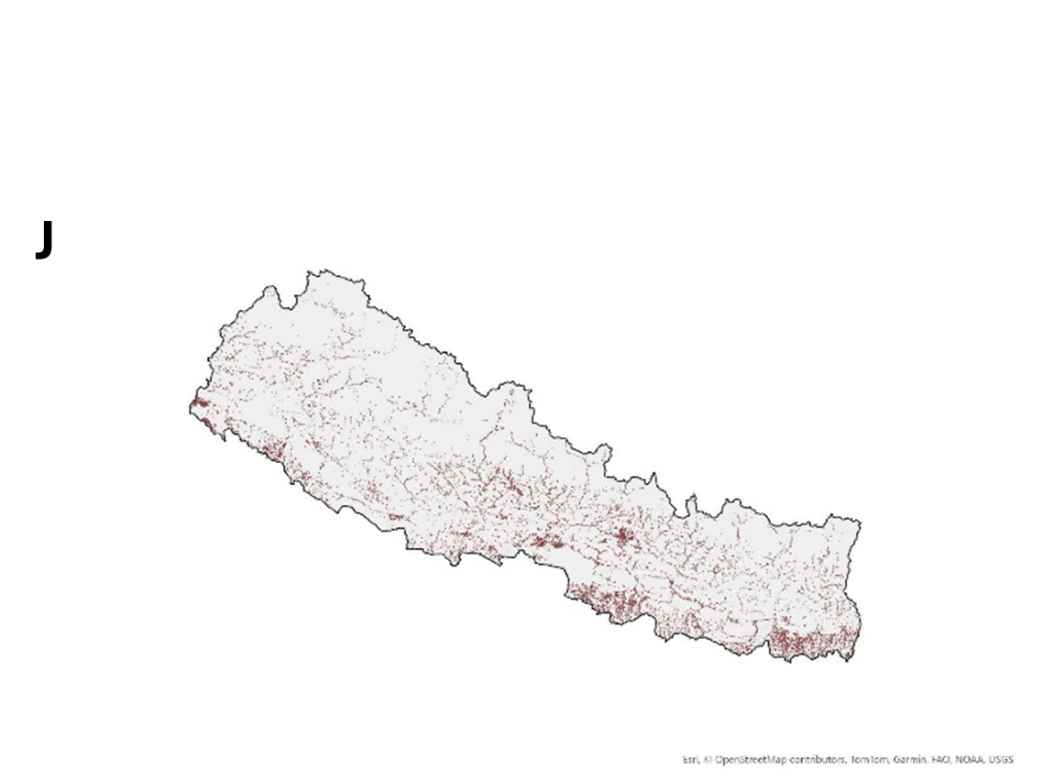


## Table 2: Emergency capacity classification criteria (adapted from Tansley et al., 2015)(3)

| **Emergency capacity level** | **Definition** | **Criteria** |  |
| --- | --- | --- | --- |
| Level A | Hospitals with 24-hour emergency services | - Overnight beds - 24-hour duty schedule - At least two qualified providers^A^ - Availability of water and electricity - Functioning telephone - On-site latrines |  |
| Level B | Hospitals with resuscitative capabilities | - Satisfies all A criteria - Availability of:   - IV fluids   - Blood products^B^   - Basic surgical instruments^C^   - Local anaesthesia   - Sterile dressings^D^   - Supplemental oxygen |  |
| Level C | Tertiary care hospitals^E^ | - *89+-0Tertiary hospital designation or any facility with all level B criteria and 50 or more inpatient beds, and surgeon on staff |  |
| Notes:   1. Qualified providers are defined as specialist physicians, medical officers, or nurses 2. The Nepal SPA 2021 survey does not have any questions about blood products 3. Basic surgical equipment refers to forceps, a needle driver, and sterile scissors 4. The Nepal SPA 2021 survey does not have any questions about sterile bandages. 5. The survey does not designate tertiary/referral hospitals, so we used the latter definition (all B criteria, 50 inpatient beds, and a surgeon on staff). | | | |

## Table 3: Nepal SPA 2021 – Facility questionnaire (NPFC8AFLSR) mapped to emergency care criteria

| Level | Criteria | Question | Question text |
| --- | --- | --- | --- |
| A | Overnight beds | V010b | Outpatient or only inpatient |
|  |  | V143 | Number of beds for overnight observation/inpatient care |
|  | 24-hour duty schedule | V503 | Schedule of 24-hour staffing |
|  |  | V101a | Presence of trained health provider at facility 24 hours with duty schedule |
|  | At least two qualified providers (specialist physicians, medical officers, or nurses) | V102a | 1 01 generalist [mdgp] 2 02 gynecologist / obstetrician 3 03 anesthesiologist 4 04 md medicine 5 05 pathologist 6 06 general surgeon 7 07 pediatrician 8 08 orthopedic 9 09 phychiatrist 10 10 radiologist 11 11 other specialists medical doctors 12 12 medical officer (mbbs) 13 13 dental officer(bds)/dental sergeon 15 14 nurse (r15) 16 15 anesthetic assistant (r14) 17 16 critical care nursing staff (trained) -r16 18 17 anm (r25) 25 18 lab technologist/officer/ lab tech/lab assistant (r17) 26 19 pharmacist (r19) 27 20 radiographer/dark room assistant (r20) 35 21 counselor with clinical qualification (htc only) (r22) 36 22 counselor without clinical qualification (htc only) (r23) 40 23 health assistant (ha) / ahw / sahw / phi (r18) 41 24 physiotherapist/physiotherapy assistant (r21) 42 25 other clinical staff not listed above (e.g., dietician) (r26) 50 26 non-clinical staff/no technical qualification (r27) 51 27 it staff (r24) |
|  | Availability of water and electricity | V120a | Facility connected to central supply electricity grid |
|  |  | V120 | Functional backup generator with fuel (or charged battery) |
|  |  | V121a | Source other than central grid: fuel-operated generator |
|  |  | V121b | Source other than central grid: battery-operated generator |
|  |  | V121c | Source other than central grid: solar system |
|  |  | V121d | Source other than central grid: invertor - cs |
|  |  | V124 | Water onsite or within 500 m of facility |
|  | Functioning telephone | V127a | Functioning landline phone in facility |
|  |  | V127b | Functioning cell phone in facility |
|  |  | V127c | Functioning short-wave radio in facility |
|  |  | V127 | Any functioning communication system observed or reported during all service tim |
|  | On-site latrines | V153 | Functioning latrine for clients (no/yes) |
|  |  | V153a | Functioning latrine for clients |
| B | Availability of iv fluids | V924_01 | Iv solution: normal saline/ sodium chloride injectable solution |
|  |  | V924_02 | Iv solution: 5% dextrose-normal saline |
|  |  | V924_03 | Iv solution: ringers lactate |
|  |  | V924_05 | Na - iv solution: cs |
|  | Local anaesthesia | V1703d | Minor surgical materials: lidocaine/ lignocaine injection |
|  |  | V1703e | Minor surgical materials: ketamine injection |
|  | Supplemental oxygen | V166p | General opd area: filled oxygen cylinder |
|  | Basic surgical instruments (forceps, a needle driver, sterile scissors) | V336g | Implant: minor surgery kit (artery forceps) |
|  |  | V1702a | Minor surgical: needle holder |
|  |  | V1702d | Minor surgical: surgical scissors |
| C | Tertiary care facilities | V143 | Number of beds for overnight observation/inpatient care (50 or more) |
|  | Surgeon on staff | V102a | 6 06 general surgeon |

## Table 4: Average speeds, all vehicle types

|  | | |  | | | Dry season | | | | | | Wet season | | | |
| --- | --- | --- | --- | --- | --- | --- | --- | --- | --- | --- | --- | --- | --- | --- | --- |
| Four-wheeler | | | | | | | | | | | | | | | |
| Class | | | Label | | | speed^A^ | | | mode | | | speed^A^ | | mode | |
| 1001 | | | Primary | | | 55 | | | MOTORIZED | | | 48.75 | | MOTORIZED | |
| 1002 | | | Secondary | | | 50 | | | MOTORIZED | | | 45 | | MOTORIZED | |
| 1003 | | | Tertiary | | | 39.38 | | | MOTORIZED | | | 30.63 | | MOTORIZED | |
| 1004 | | | Minor | | | 25 | | | MOTORIZED | | | 23.33 | | MOTORIZED | |
| 2 | | | Tree cover | | | 3.2 | | | WALKING | | | 3.2 | | WALKING | |
| 11 | | | Shrubland/Grassland | | | 4.2 | | | WALKING | | | 3.6 | | WALKING | |
| 5 | | | Cropland | | | 3.2 | | | WALKING | | | 4 | | WALKING | |
| 7 | | | Built-up | | | 5 | | | WALKING | | | 5 | | WALKING | |
| 8 | | | Bare sparse vegetation | | | 3 | | | WALKING | | | 3 | | WALKING | |
| 9 | | | Snow and Ice | | | 1.6 | | | WALKING | | | 1.6 | | WALKING | |
| 1 | | | Permanent water bodies | | | 0 | | | WALKING | | | 0 | | WALKING | |
| 4 | | | Herbaceous wetland | | | 0.5 | | | WALKING | | | 0.5 | | WALKING | |
| 1005 | | | Minor_pedestrian | | | 4.3 | | | WALKING | | | 3.7 | | WALKING | |
| 10000 | | | Bridge | | | 4.3 | | | WALKING | | | 3.7 | | WALKING | |
| 100000 | | | River | | | 0 | | | WALKING | | | 0 | | WALKING | |
|  | | |  | | |  | | |  | | |  | |  | |
| Two-wheeler | | | | | | | | | | | | | | | |
|  | |  | | | Dry season | | | | | | Wet season | | | | |
| Class | | Label | | | speed | | | mode | | | speed | | | mode | |
| 1001 | | Primary | | | 55 | | | MOTORIZED | | | 45 | | | MOTORIZED | |
| 1002 | | Secondary | | | 48.8 | | | MOTORIZED | | | 40 | | | MOTORIZED | |
| 1003 | | Tertiary | | | 42.5 | | | MOTORIZED | | | 33.8 | | | MOTORIZED | |
| 1004 | | Minor | | | 24.29 | | | MOTORIZED | | | 15 | | | MOTORIZED | |
| 2 | | Tree cover | | | 3.2 | | | WALKING | | | 2.4 | | | WALKING | |
| 11 | | Shrubland/Grassland | | | 3.6 | | | WALKING | | | 2.7 | | | WALKING | |
| 5 | | Cropland | | | 4 | | | WALKING | | | 3.15 | | | WALKING | |
| 7 | | Built-up | | | 5 | | | WALKING | | | 3.7 | | | WALKING | |
| 8 | | Bare sparse vegetation | | | 3 | | | WALKING | | | 2.2 | | | WALKING | |
| 9 | | Snow and Ice | | | 1.6 | | | WALKING | | | 1.2 | | | WALKING | |
| 1 | | Permanent water bodies | | | 0 | | | WALKING | | | 0 | | | WALKING | |
| 4 | | Herbaceous wetland | | | 0.5 | | | WALKING | | | 0 | | | WALKING | |
| 10000 | | Bridge | | | 30 | | | MOTORIZED | | | 23.8 | | | MOTORIZED | |
| 100000 | | River | | | 0 | | | WALKING | | | 0 | | | WALKING | |
| 1001 | | Primary | | | 55 | | | MOTORIZED | | | 45 | | | MOTORIZED | |
|  | |  | | |  | | |  | | |  | | |  | |
| Three-wheeler | | | | | | | | | | | | | | | |
|  |  | | | Dry season | | | | | | Wet season | | | | |  |
| Class | Label | | | speed | | | mode | | | speed | | | mode | |  |
| 1001 | Primary | | | 37.5 | | | MOTORIZED | | | 33 | | | MOTORIZED | |  |
| 1002 | Secondary | | | 35 | | | MOTORIZED | | | 29 | | | MOTORIZED | |  |
| 1003 | Tertiary | | | 30 | | | MOTORIZED | | | 24 | | | MOTORIZED | |  |
| 1004 | Minor | | | 26.67 | | | MOTORIZED | | | 18 | | | MOTORIZED | |  |
| 2 | Tree cover | | | 3.2 | | | WALKING | | | 2.4 | | | WALKING | |  |
| 11 | Shrubland/Grassland | | | 4.2 | | | WALKING | | | 3.15 | | | WALKING | |  |
| 5 | Cropland | | | 3.2 | | | WALKING | | | 2.7 | | | WALKING | |  |
| 7 | Built-up | | | 5 | | | WALKING | | | 3.73 | | | WALKING | |  |
| 8 | Bare sparse vegetation | | | 3 | | | WALKING | | | 2.2 | | | WALKING | |  |
| 9 | Snow and Ice | | | 1.6 | | | WALKING | | | 1.2 | | | WALKING | |  |
| 1 | Permanent water bodies | | | 0 | | | WALKING | | | 0 | | | WALKING | |  |
| 4 | Herbaceous wetland | | | 0.5 | | | WALKING | | | 0 | | | WALKING | |  |
| 1005 | Minor_pedestrian | | | 4.3 | | | WALKING | | | 3.7 | | | WALKING | |  |
| 10000 | Bridge | | | 4.3 | | | WALKING | | | 3.7 | | | WALKING | |  |
| 100000 | River | | | 0 | | | WALKING | | | 0 | | | WALKING | |  |
|  |  | | |  | | |  | | |  | | |  | |  |
| 1. Speeds are based on Ochoa et al. 2023 and simplified to fewer road classes (2) | | | | | | | | | | | | | | |  |

## Table 5: Sample characteristics for injured people

|  | N | % |
| --- | --- | --- |
| Total | 1,342 | 100 |
| Sex |  |  |
| Male | 819 | 61.0 |
| Female | 523 | 39.0 |
| Age category |  |  |
| <15 | 224 | 16.7 |
| 15-24 | 202 | 15.1 |
| 25-34 | 218 | 16.2 |
| 35-44 | 205 | 15.3 |
| 45-59 | 272 | 20.3 |
| 60+ | 182 | 13.6 |
| Type of incident |  |  |
| Road traffic collision | 525 | 40.3 |
| Other (not specified) | 778 | 59.7 |
| Type of vehicle^A^ |  |  |
| Car | 17 | 3.3 |
| Truck/Bus | 44 | 8.4 |
| Motorcycle | 348 | 66.8 |
| Bicycle | 60 | 11.5 |
| Pedestrian | 27 | 5.2 |
| Other vehicle | 25 | 4.8 |
| Complication^B^ |  |  |
| Any disability | 695 | 51.8 |
| Severe disability | 192 | 14.3 |
| Death | 24 | 1.8 |
| Type of injury |  |  |
| Broken bone | 389 | 29.6 |
| Burn | 18 | 1.4 |
| Cut/bite/open wound | 590 | 44.9 |
| Head injury | 28 | 2.1 |
| Internal injury | 83 | 6.3 |
| Poisoning | 4 | 0.3 |
| Spinal cord injury | 7 | 0.5 |
| Suffocation or asphyxiation | 199 | 15.1 |
| Type of disability^,C^ |  |  |
| Brain damage | 4 | 0.6 |
| Chronic pain | 467 | 50.3 |
| Emotional trauma | 6 | 0.6 |
| Loss of eyesight or hearing | 5 | 0.5 |
| Loss of limb, paralyzed, or disfigurement | 8 | 0.8 |
| Loss of limb function | 89 | 9.6 |
| Multiple disabilities | 100 | 10.8 |
| Other | 16 | 1.7 |
| Notes:   1. This question is only asked for those who report a road traffic collision (n=525), with 4 missing responses. 2. Any disability is defined as paralyzed, brain damage, disfigurement, loss of limb, loss of limb function, loss of eyesight, loss of hearing, chronic pain, emotional trauma, other. Severe disability is defined as paralyzed, brain damage, disfigurement, loss of limb, loss of limb function, loss of eyesight, loss of hearing 3. More than one response is possible (e.g., a disability and a severe disability) | | |

##

## Table 6: High-resolution physical access to government hospitals in under two hours

|  |  | Level A | | | | Level B | | | | Level C | | | |
| --- | --- | --- | --- | --- | --- | --- | --- | --- | --- | --- | --- | --- | --- |
| Level | Geography | All DHS households  N=13,908 | | DHS households reporting injury  N=1,426 | | All DHS households  N=13,908 | | DHS households reporting injury  N=1,426 | | All DHS households  N=13,908 | | DHS households reporting injury  N=1,426 | |
|  |  | n | % | n | % | n | % | n | % | n | % | n | % |
| National | Nepal | 12689 | 91.2 | 1305 | 91.5 | 12455 | 89.6 | 1273 | 89.3 | 9788 | 70.4 | 1002 | 70.3 |
| Province | Koshi | 1934 | 89.1 | 177 | 89.8 | 1904 | 87.7 | 177 | 89.8 | 1410 | 64.9 | 1002 | 69.0 |
|  | Madhesh | 2069 | 100 | 234 | 100.0 | 2069 | 100.0 | 234 | 100.0 | 2069 | 100.0 | 234 | 100.0 |
|  | Bagmati | 2225 | 97.3 | 230 | 97.9 | 2225 | 97.3 | 230 | 97.9 | 1649 | 72.1 | 165 | 70.2 |
|  | Gandaki | 1707 | 94.1 | 128 | 92.8 | 1678 | 92.5 | 126 | 91.3 | 1499 | 82.6 | 114 | 82.6 |
|  | Lumbini | 1955 | 97.1 | 200 | 98.0 | 1927 | 95.7 | 196 | 96.1 | 1841 | 91.5 | 194 | 95.1 |
|  | Karnali | 1285 | 72.6 | 164 | 71.9 | 1138 | 64.3 | 138 | 60.5 | 270 | 15.2 | 28 | 12.3 |
|  | Sudurpashchim | 1514 | 84.9 | 172 | 90.5 | 1514 | 84.9 | 172 | 90.5 | 1050 | 58.9 | 131 | 68.9 |
| Ecological zone | Terai | 6461 | 87.5 | 664 | 87.4 | 6257 | 84.8 | 632 | 83.2 | 4660 | 63.1 | 467 | 61.4 |
|  | Hills | 4119 | 98.5 | 450 | 98.9 | 4119 | 98.5 | 450 | 98.9 | 3687 | 88.2 | 398 | 87.5 |
|  | Mountains | 1934 | 89.1 | 177 | 89.8 | 1904 | 87.7 | 177 | 177 | 1410 | 1410 | 136 | 69.0 |
| Type of area | Urban | 6969 | 95.9 | 714 | 95.2 | 6909 | 95.1 | 709 | 94.5 | 5768 | 79.4 | 577 | 76.9 |
|  | Rural | 5720 | 86.1 | 591 | 87.4 | 5546 | 83.5 | 564 | 83.4 | 4020 | 60.5 | 425 | 62.9 |
| Wealth index^A^ | Quintile 1 | 3211 | 76.3 | 324 | 77.0 | 3055 | 72.6 | 324 | 77.0 | 1599 | 38.0 | 159 | 37.8 |
|  | Quintile 2 | 2718 | 95.1 | 270 | 94.7 | 2663 | 93.2 | 270 | 94.7 | 2088 | 73.1 | 210 | 73.7 |
|  | Quintile 3 | 2598 | 98.4 | 295 | 98.3 | 2582 | 97.8 | 295 | 98.3 | 2330 | 88.2 | 260 | 86.7 |
|  | Quintile 4 | 2353 | 99.1 | 234 | 99.6 | 2347 | 98.8 | 234 | 99.6 | 2120 | 89.3 | 212 | 90.2 |
|  | Quintile 5 | 1809 | 99.1 | 182 | 98.4 | 1808 | 99.0 | 182 | 98.4 | 1651 | 90.4 | 161 | 87.0 |
| Climate change vulnerability | High or very high | 2121 | 79.0 | 252 | 82.1 | 2031 | 75.1 | 236 | 76.9 | 992 | 36.7 | 118 | 38.4 |
|  | Moderate | 5745 | 91.3 | 576 | 91.0 | 5601 | 89.0 | 560 | 88.5 | 4267 | 67.8 | 432 | 68.2 |
|  | Low or very low | 4674 | 98.2 | 453 | 98.1 | 4674 | 98.2 | 453 | 98.1 | 4380 | 92.0 | 428 | 92.6 |

## Figure K: Map of topography, provinces, ecological zones, sampled households, and households reporting injury in the past year


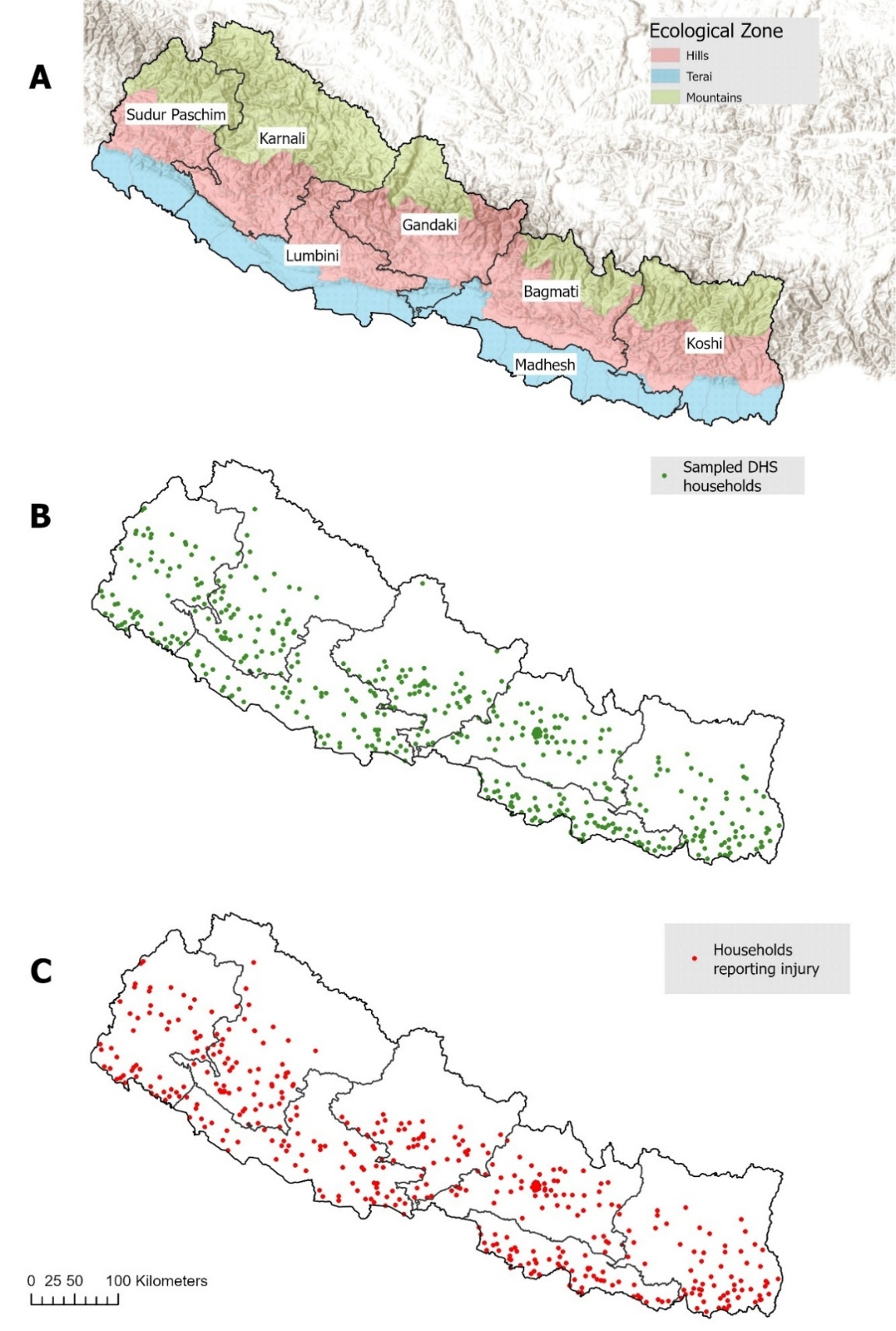


Caption: A: Topographic map of Nepal with regions and ecological zones; B: Locations of sampled households for the Nepal DHS 2022; C: Locations of sampled households reporting injury in the past year in the Nepal DHS 2022

## Figure L: Maps of the spatial distribution of Level A, B, and C government hospitals


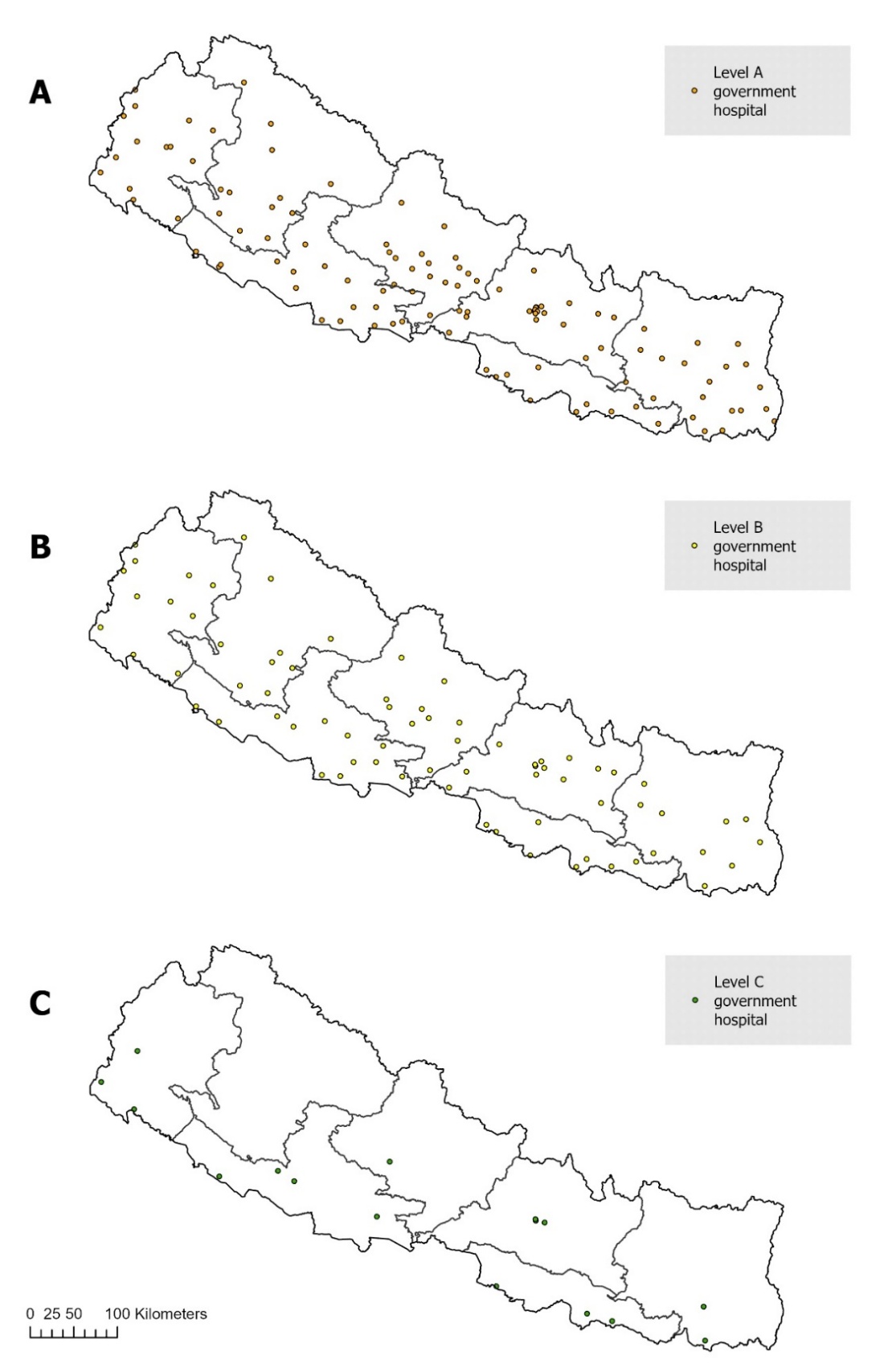


Caption: A: Map of Level A emergency care (hospitals with 24-hour emergency services) from the DHS SPA 2021; B: Map of Level B emergency care (hospitals with resuscitative capabilities) from the DHS SPA 2021; C: Map of Level C emergency care ( ‘tertiary’ hospitals) from the DHS SPA 2021

##
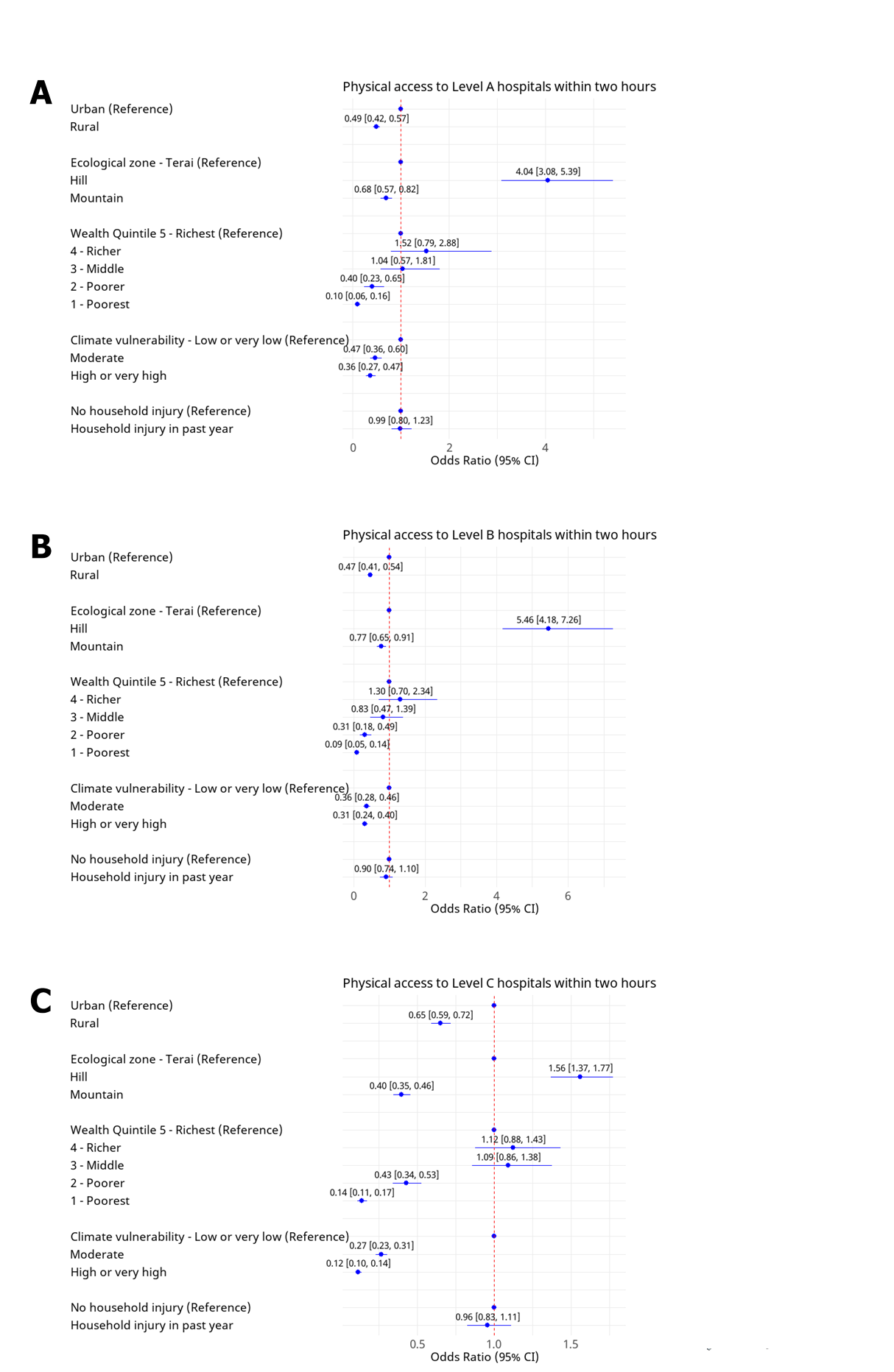
Figure M: Logistic regression results for two-hour access to government hospitals

Caption: A: Two-hour access to Level A emergency care (hospitals with 24-hour emergency services); B: Two-hour access to Level B emergency care (hospitals with resuscitative capabilities); C: Two-hour access to Level C emergency care (‘tertiary’ hospitals)

## References

1. Mainali J, Pricope NG. High-resolution spatial assessment of population vulnerability to climate change in Nepal. Applied Geography. 2017;82:66-82.

2. Ochoa C, Rai M, Martins SB, Alcoba G, Bolon I, de Castañeda RR, et al. Vulnerability to snakebite envenoming and access to healthcare in the Terai region of Nepal: a geospatial analysis. The Lancet Regional Health-Southeast Asia. 2023;9.

3. Tansley G, Schuurman N, Amram O, Yanchar N. Spatial access to emergency services in low-and middle-income countries: a GIS-based analysis. PloS one. 2015;10(11):e0141113.
