# Supplementary material for: Climate, geography and socioeconomic vulnerability and emergency care accessibility in Nepal, 2022: a high-resolution geospatial analysis of inequalities
Source: BMJ Glob Health. 2026 Apr 30;11(4):e021150. doi: 10.1136/bmjgh-2025-021150 (PMC13141136; doi:10.1136/bmjgh-2025-021150)
Supplement: online supplemental file 2 [file bmjgh-11-4-s002.docx]

### BMJ Global Health Author Reflexivity Statement

Adapted from Morton, B., Vercueil, A., Masekela, R., Heinz, E., Reimer, L., Saleh, S., Kalinga, C., Seekles, M., Biccard, B., Chakaya, J., Abimbola, S., Obasi, A. and Oriyo, N. (2022), Consensus statement on measures to promote equitable authorship in the publication of research from international partnerships. Anaesthesia, 77: 264-276. <https://doi.org/10.1111/anae.15597>

| **Study conceptualisation** | |
| --- | --- |
| 1. How does this study address local research and policy priorities? | Nepal has committed to strengthening its emergency care system, including investment in ambulance services and burn and trauma centres. This is an important research/policy area as Nepal faces a high injury burden and is one of the most climate-vulnerable and disaster-prone countries in the world. This study directly relates to this goal by comprehensively estimating emergency care coverage and identifying where there are unmet needs. |
| 1. How were local researchers involved in study design? | Nepal-based and international co-authors jointly shaped the research questions and analytical focus, drawing on local clinical, health systems, and policy expertise to ensure relevance to the Nepalese context. |
| **Research management** | |
| 1. How has funding been used to support the local research team(s)? | One Nepal-based researcher was supported through funding from the National Institutes of Health Fogarty International Center. Other co-authors contributed without dedicated funding for this project. |
| **Data acquisition and analysis** | |
| 1. How are research staff who conducted data collection acknowledged? | This study exclusively used publicly available secondary data; no primary data collection was undertaken. |
| 1. How have members of the research partnership been provided with access to study data? | All data sources were publicly accessible to all members of the research team. |
| 1. How were data used to develop analytical skills within the partnership? | The analysis was led by the first author (AM), an early-career researcher, with collaborative input from co-authors on study design, analytical decisions, and interpretation. This process facilitated skills exchange across the partnership. |
| **Data interpretation** | |
| 1. How have research partners collaborated in interpreting study data? | All authors interpreted the study findings, with Nepalese research partners providing critical insights into the context, including on the evolution of the emergency care system and structural inequalities in care due to country-specific factors (e.g., topography, socioeconomic conditions). |
| **Drafting and revising for intellectual content** | |
| 1. How were research partners supported to develop writing skills? | All authors were invited to contribute to drafting and revising the manuscript, with iterative feedback provided throughout the writing process. |
| 1. How will research products be shared to address local needs? | Findings will be shared with Nepal-based collaborators and professional networks. Results will be presented at national and international meetings, with the aim of informing ongoing discussions on emergency care planning and policy in Nepal. |
| **Authorship** | |
| 1. How is the leadership, contribution and ownership of this work by LMIC researchers recognised within the authorship? | LMIC researchers are included as co-authors, with author order reflecting the nature and extent of individual contributions in line with journal authorship criteria. |
| 1. How have early career researchers across the partnership been included within the authorship team? | Several authors (AM, AD, CO, RS) are early-career researchers and were actively involved in study design, analysis, interpretation, and manuscript preparation. |
| 1. How has gender balance been addressed within the authorship? | The authorship team includes three women (AM, AD, RS). |
| **Training** | |
| 1. How has the project contributed to training of LMIC researchers? | The project supported meaningful research engagement for a Nepal-based surgeon in training (RS), including contribution to study interpretation and authorship, thereby strengthening research capacity in emergency care. |
| **Infrastructure** | |
| 1. How has the project contributed to improvements in local infrastructure? | While not directly focused on infrastructure development, the study contributed to strengthening individual research competencies, including data interpretation and academic writing, which support longer-term institutional research capacity. |
| **Governance** | |
| 1. What safeguarding procedures were used to protect local study participants and researchers? | No safeguarding procedures were required as the study used publicly available, de-identified secondary data. |
